# Nitrogenase cofactor biosynthesis using proteins produced in mitochondria of *Saccharomyces cerevisiae*

**DOI:** 10.1128/mbio.03088-23

**Published:** 2023-12-21

**Authors:** Katarzyna Dobrzyńska, Ana Pérez-González, Carlos Echavarri-Erasun, Diana Coroian, Alvaro Salinero-Lanzarote, Marcel Veldhuizen, Dennis R. Dean, Stefan Burén, Luis M. Rubio

**Affiliations:** 1 Centro de Biotecnología y Genómica de Plantas, Universidad Politécnica de Madrid (UPM), Instituto Nacional de Investigación y Tecnología Agraria y Alimentaria (INIA/CSIC), Pozuelo de Alarcón, Spain; 2 Departamento de Biotecnología-Biología Vegetal, Escuela Técnica Superior de Ingeniería Agronómica, Alimentaría y de Biosistemas, Universidad Politécnica de Madrid, Madrid, Spain; 3 Department of Biochemistry, Virginia Tech, Blacksburg, Virginia, USA; University of California, Berkeley, California, USA

**Keywords:** NifEN, FeMo-co biosynthesis, mitochondria, nitrogen fixation, pathway engineering, yeast, *Azotobacter vinelandii*

## Abstract

Biological nitrogen fixation, the conversion of inert N_2_ to metabolically tractable NH_3_, is only performed by certain microorganisms called diazotrophs and is catalyzed by the nitrogenases. A [7Fe-9S-C-Mo-*R*-homocitrate]-cofactor, designated FeMo-co, provides the catalytic site for N_2_ reduction in the Mo-dependent nitrogenase. Thus, achieving FeMo-co formation in model eukaryotic organisms, such as *Saccharomyces cerevisiae*, represents an important milestone toward endowing them with a capacity for Mo-dependent biological nitrogen fixation. A central player in FeMo-co assembly is the scaffold protein NifEN upon which processing of NifB-co, an [8Fe-9S-C] precursor produced by NifB, occurs. Prior work established that NifB-co can be produced in *S. cerevisiae* mitochondria. In the present work, a library of *nifEN* genes from diverse diazotrophs was expressed in *S. cerevisiae*, targeted to mitochondria, and surveyed for their ability to produce soluble NifEN protein complexes. Many such NifEN variants supported FeMo-co formation when heterologously produced in the diazotroph *A. vinelandii*. However, only three of them accumulated in soluble forms in mitochondria of aerobically cultured *S. cerevisiae*. Of these, two variants were active in the *in vitro* FeMo-co synthesis assay. NifEN, NifB, and NifH proteins from different species, all of them produced in and purified from *S. cerevisiae* mitochondria, were combined to establish successful FeMo-co biosynthetic pathways. These findings demonstrate that combining diverse interspecies nitrogenase FeMo-co assembly components could be an effective and, perhaps, the only approach to achieve and optimize nitrogen fixation in a eukaryotic organism.

**IMPORTANCE:**

Biological nitrogen fixation, the conversion of inert N2 to metabolically usable NH3, is a process exclusive to diazotrophic microorganisms and relies on the activity of nitrogenases. The assembly of the nitrogenase [7Fe-9S-C-Mo-*R*-homocitrate]-cofactor (FeMo-co) in a eukaryotic cell is a pivotal milestone that will pave the way to engineer cereals with nitrogen fixing capabilities and therefore independent of nitrogen fertilizers. In this study, we identified NifEN protein complexes that were functional in the model eukaryotic organism *Saccharomyces cerevisiae*. NifEN is an essential component of the FeMo-co biosynthesis pathway. Furthermore, the FeMo-co biosynthetic pathway was recapitulated *in vitro* using only proteins expressed in *S. cerevisiae*. FeMo-co biosynthesis was achieved by combining nitrogenase FeMo-co assembly components from different species, a promising strategy to engineer nitrogen fixation in eukaryotic organisms.

## INTRODUCTION

Nitrogen fixation is the process that converts inert dinitrogen (N_2_) into metabolically tractable ammonia (NH_3_). It occurs biologically only in microorganisms called diazotrophs but also industrially through the Haber-Bosch process (1). The industrial process is extremely resource intensive having severe negative environmental impacts. Nevertheless, synthetic fertilizers have proven fundamental for increasing the crop productivity that has fueled growth of the human population over the past 100 years (2). With increasing evidence of climate change and the vast inequalities between farming in the developed and developing countries, there is an urgent need to develop more sustainable ways to support agriculture through production of nitrogen fertilizers (3, 4). One such approach involves transferring active forms of the genetic determinants of biological nitrogen fixation from microbial diazotrophs to plants, and this goal has represented an unrealized challenge to the scientific community ever since the emergence of recombinant DNA technology. The success of these efforts has been denied owing to both the biochemical and genetic complexity of the biological process as well as the susceptibility of the catalytic components and certainty of their assembly factors to oxidative inactivation (5–7). However, now that many of the basic genetic and biochemical features of microbial biological nitrogen fixation are understood, together with demonstration that the oxygen sensitivity issue can be resolved by subcellular targeting, there has been a renewed interest in efforts to endow eukaryotes with a capacity for biological nitrogen fixation (8).

The only known catalysts for biological nitrogen fixation are the nitrogenases. So far, three genetically distinct but mechanistically similar nitrogenase isoforms have been described. Among the nitrogenase isoforms, the Mo-dependent enzyme, which is produced by all diazotrophs, has the highest catalytic efficiency making it a favored target for ultimate expression in crop plants (9). Mo-dependent nitrogenase is a complex two-component metalloenzyme. The two components include MoFe protein (or dinitrogenase) and Fe protein (or dinitrogenase reductase). Hereafter, the Mo-dependent nitrogenase catalytic components, as well as associated proteins involved in their activation, will be designated according to the corresponding genes encoding them. Hence, MoFe protein, encoded by *nifDK*, and Fe protein, encoded by *nifH*, are designated NifDK and NifH, respectively . The homodimeric NifH contains a [4Fe-4S] cluster which serves as an agent of nucleotide-dependent electron delivery to NifDK (10), a heterotetramer that provides the site for N_2_ binding and reduction. There are two complex metalloclusters contained in NifDK, an [7Fe-9S-C-Mo-*R*-homocitrate]-cofactor (FeMo-co) and an [8Fe-7S] P-cluster. The P-cluster is involved in intercomponent electron transfer whereas FeMo-co provides the site for N_2_ binding and reduction (11, 12). In addition to its catalytic role involving N_2_ reduction, NifH is also required for the assembly of both FeMo-co and P-clusters (13).

**Fig 1:**
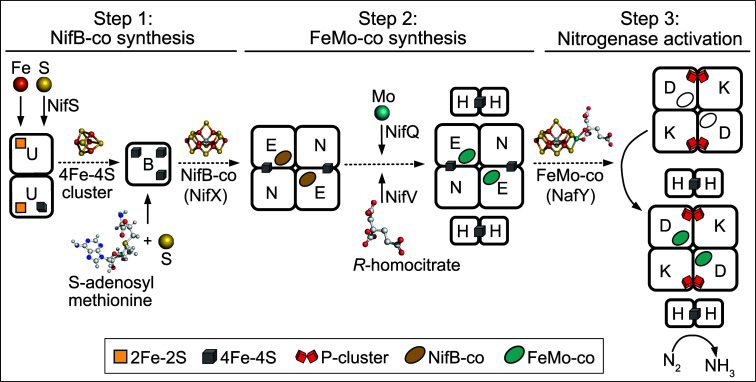
Simplified representation of FeMo-co synthesis and its insertion into the apo-form of NifDK. The process can be considered to occur in three steps. In step 1, transient [4Fe-4S] clusters are assembled on the NifU scaffold and delivered to NifB. There are two permanent [2Fe-2S] clusters contained within the NifU homodimer involved in the formation of transient [4Fe-4S] formed on the NifU scaffold. The S necessary for formation of the transient [4Fe-4S] clusters is provided by the pyridoxal phosphate-dependent enzyme NifS. NifB can harbor three [4Fe-4S] clusters. One of these is a permanent cluster that can bind *S*-adenosylmethionine (SAM). The two other clusters are fused to form NifB-co which also involves incorporation of the central carbon provided by the 5-methyl group of SAM. NifB-co is subsequently delivered to the NifEN scaffold which contains two permanent [4Fe-4S] clusters. In step 2, NifB-co is converted to FeMo-co by replacement of an apical Fe atom within NifB-co by Mo and attachment of the organic constituent *R*-homocitrate in a process that involves NifV (homocitrate synthase), NifQ to supply Mo, and NifH. In the final step, FeMo-co is delivered to an apo-form of NifDK that contains P-clusters but not FeMo-co. Mature NifDK, together with NifH, catalyzes the nucleotide-dependent reduction of N_2_ to yield NH_3_. The interaction of NifH with the NifEN scaffold during FeMo-co formation and the interaction of NifH with NifDK during catalysis is transient. In *A. vinelandii*, NifX and NafY are involved in the transfer of NifB-co from NifB to NifEN and FeMo-co from NifEN to NifDK, respectively. Atoms are represented as follows: orange, iron; yellow, sulfur; gray, carbon; red, oxygen; turquoise, molybdenum; blue, nitrogen. The [4Fe-4S] cluster structure was extracted from PDB 2NIP. FeMo-co and *R*-homocitrate structures were extracted from PDB 3U7Q. NifB-co structure is derived from FeMo-co with Fe replacing Mo and lacking *R*-homocitrate. The SAM structure was obtained from the RCSB Protein Data Bank. Structures were generated with PyMol (Schrödinger).

Activation of a form of NifDK, designated apo-NifDK, that contains intact P-clusters but not FeMo-co requires the separate formation and subsequent insertion of FeMo-co. Consequently, an ability to produce FeMo-co in model eukaryotes is an essential aspect of endowing them with a capacity for nitrogen fixation. Eight different *nif* gene products are involved in FeMo-co assembly, schematically shown in Fig. 1 (13, 14). The key to this process is NifEN, structurally similar to the homologous NifDK protein, that provides a heterotetrameric scaffold for the assembly of FeMo-co from an [8Fe-9S-C] precursor designated NifB-co. This process involves Mo substitution for an apical Fe atom within NifB-co and attachment of the organic constituent, homocitrate, to the Mo atom. Prior work demonstrated NifB-co can be produced in *Saccharomyces cerevisiae* mitochondria thereby making NifEN an important next target protein for endowing a model eukaryote with an ability to produce FeMo-co (15). In this regard, NifEN from *Paenibacillus polymyxa* WLY78 and *Klebsiella oxytoca* were previously expressed in yeast cytosol and tobacco mitochondria, respectively, but no soluble protein complex was reported (16, 17). In tobacco, the expression of the *K. oxytoca* NifE polypeptide only accumulated in the insoluble fraction (17). Also, the attempts to produce *Azotobacter vinelandii* NifEN in *S. cerevisiae* mitochondria generated a species that migrated as a single band when analyzed by SDS-PAGE indicating that a functional NifEN complex was probably not produced (18).

Given the advances in synthetic biology and prior success in producing active forms of NifH and NifB in model eukaryotic mitochondria (15, 19–25), we chose to perform a phylogenetic survey for the production of active forms of NifEN targeted to *S. cerevisiae* mitochondria. Toward this end, a library consisting of 17 different NifEN protein variants encoded by a variety of phylogenetically diverse diazotrophs were produced and tested for *in vivo* functionality in the model prokaryotic diazotroph *A. vinelandii*. The same library was expressed in *S. cerevisiae*, targeted to mitochondria, and evaluated for accumulation of soluble NifEN complexes. Selected NifEN constructs that were active in *A. vinelandii* and formed soluble complexes in mitochondria of *S. cerevisiae* were isolated from the latter and evaluated in the *in vitro* FeMo-co synthesis assay.

## RESULTS

### Selection of diverse naturally occurring NifEN protein variants

A library containing 17 *nifEN* gene cassettes was prepared from diverse bacterial and archaeal diazotrophs. Selection of NifEN candidates was biased toward: (i) organisms having an established ability to fix nitrogen, (ii) thermophilic organisms that might harbor NifEN having the ability to withstand the elevated temperatures of the mitochondria (26), (iii) aerobic and aerobic photosynthetic organisms that might produce more O_2_-resistant NifEN variants, and (iv) organisms that were shown in previous studies to be sources of soluble NifH and NifB when expressed in *S. cerevisiae* or other eukaryotes (Table S1) (15, 20–23, 25). Two organisms, *Anabaena variabilis* and *Geobacter metallireducens*, that produce natural “NifEN fusion proteins” were also selected because previous work by Allen and colleagues showed that a synthetic gene fusion between *nifD* and *nifK* generated a NifDK protein that is soluble and more resistant to degradation when expressed in *Nicotiana benthamiana* mitochondria (27). All NifEN candidates are similar based on primary structure comparisons and contain conserved cysteine residues known or suspected to coordinate NifEN-associated metalloclusters (Fig. S1) (28). All coding regions for gene cassettes were synthesized using codon-usage bias optimized for *S. cerevisiae*.

### Functional screening of diverse NifEN variants in *A. vinelandii*

**Fig 2:**
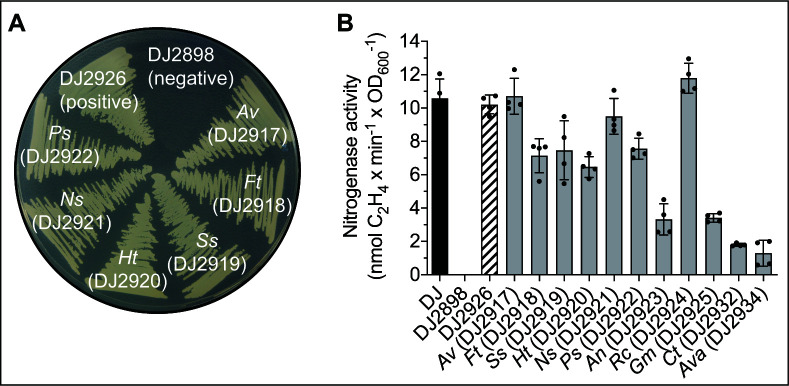
*In vivo* functionality of the NifEN candidates *in A. vinelandii*. (**A**) Diazotrophic growth of *A. vinelandii* DJ2898 transformed with six different *nifEN* gene cassettes. Performance of all the strains transformed with the different *nifEN* variants are shown in Fig. S2. (**B**) *In vivo* acetylene reduction activities of strains with restored Nif+ phenotype. DJ2926 results from transformation of DJ2898 with the native *A. vinelandii nifEN* genes. Error bars indicate mean activity ± SD; *n* = 2 biological replicates shown by dots (two technical replicates each). Note that the individual activity values contributing to the mean were identical for DJ2926 and DJ2932 (*Ct*). *Av*, *A. vinelandii*; *Ft*, *F. thermalis*; *Ss*, *Synechococcus* sp. JA-2–3B’a; *Ht*, *H. thermophilus*; *Ns*, *Nostoc* sp*.*; *Ps*, *P. sabinae*; *An*, *A. nitrofigilis*; *Rc*, *R. capsulatus*; *Gm*, *G. metallireducens*; *Ct*, *C. tepidum*; *Ava*, *A. variabilis*. Refer to Table S2 and the Materials and Methods section found in the SI for further details.

Screening of NifEN candidates was performed in parallel using *A. vinelandii* and *S. cerevisiae* as heterologous host organisms. In the case of *A. vinelandii*, *in vivo* functionality and compatibility with other components of the host FeMo-co biosynthetic pathway was tested using mutant strain DJ2898 which is incapable of Mo-dependent diazotrophic growth because it carries a deletion for the endogenous *nifEN* genes. To facilitate isolation of the NifEN protein, a nucleotide sequence encoding a Single-Strep affinity-tag (“SS-tag”) was placed before the *nifE* start codon. Placement of the SS-tag at the N-terminal location was selected because *A. vinelandii* can tolerate N-terminal extensions of NifE without affecting its expression or the activity of NifEN (29). The 17 *nifEN* gene cassettes constructed in this way were integrated into the sucrose catabolic gene region (*scr*) of the *A. vinelandii* genome, and their expression was controlled by the *Escherichia coli* arabinose regulatory elements, similarly to the approach previously reported by Dos Santos and colleagues (30). Eleven of the 17 species-specific *nifEN* constructs could rescue the null diazotrophic growth phenotype of DJ2898 when arabinose was present in the growth medium (Fig. 2A; Fig. S2; Table S2). The function of the heterologously produced NifEN proteins was further verified in the rescued strains by using the *in vivo* acetylene reduction assay (31) as a proxy for N_2_ reduction (Fig. 2B).

### Subunit solubility and complex formation of NifEN variants produced in *S. cerevisiae* mitochondria

**Fig 3:**
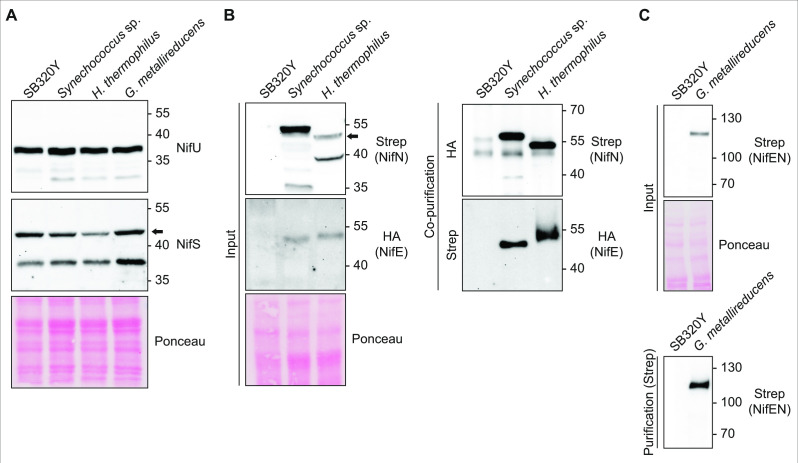
Accumulation of soluble NifEN proteins in *S. cerevisiae*. (**A**) Immunoblots detecting *Sc*NifU*^Av^* and *Sc*NifS*^Av^* proteins in soluble extracts from SB320Y (yeast strain used to express the *nifEN* library) and from yeast strains expressing the indicated NifEN variants. The black arrow points to full-length NifS (51.3 kDa). (**B, C**) Immunoblot analysis of NifEN co-purification (**B**) or purification (**C**) experiments and soluble cell-free extracts (“input”). (**B**) SS-tagged *Sc*NifN*^Ss^* and *Sc*NifN*^Ht^* were detected in the eluate from purifications targeting the HA-tag at *Sc*NifE*^Ss^* and *Sc*NifE*^Ht^*, respectively. HA-tagged *Sc*NifE*^Ss^* and *Sc*NifE*^Ht^* were detected in the eluate from purifications targeting the SS-tag at *Sc*NifN*^Ss^* and *Sc*NifN*^Ht^*, respectively. The black arrow points to full-length *Sc*NifN*^Ht^*. (**C**) TS-tagged *Sc*NifEN*^Gm^* was detected in the eluate from purifications targeting the TS-tag. Molecular weight markers (kDa) and antibodies used are indicated.

In the case of *S. cerevisiae*, the solubility of the 17 diverse NifEN subunits when expressed and targeted to *S. cerevisiae* mitochondria, as well as their capacity for NifEN complex formation, was investigated by using affinity purification tags attached to either NifE or NifN. In this way, solubility and co-purification of NifN could be evaluated using an affinity purification tag located on NifE and vice versa. The various *nifE*, *nifN*, and *nifEN* fusion genes were cloned into expression vectors for galactose-inducible expression in aerobically cultured *S. cerevisiae* cells (Table S3). The heterologously expressed NifE proteins contain an N-terminal HA-tag, and the NifN proteins contain a C-terminal SS-tag. Individual *nifE* and *nifN* genes were co-expressed from pESC vectors using GAL10 promoters to produce NifE and NifN at similar levels, and both were targeted to the *S. cerevisiae* mitochondria using the SU9 pre-sequence (Table S3) (32). As only a single tag was required for NifEN fusion proteins originating from *A. variabilis* and *G. metallireducens*, these variants were instead expressed with a Twin-Strep-tag II (“TS-tag”) affinity purification sequence located at the N-terminus of NifEN. All NifEN proteins were produced in a *S. cerevisiae* background strain that also expresses mitochondria-targeted NifU and NifS from *A. vinelandii* (Fig. S3A). It was previously shown that *A. vinelandii* NifU and NifS, which could be required to supply the [4Fe-4S] clusters contained in the NifEN scaffold, are active when produced in *S. cerevisiae* (15). While most *Sc*NifE and *Sc*NifN proteins could be detected in total protein extracts, many variants generated proteins exhibiting migration patterns on SDS-PAGE different than otherwise predicted (Fig. S3B through D). This could be due to degradation of subunits that were not properly assembled into a functional heterotetramer or from polypeptides that were not correctly targeted. Such mutual stability has been reported for NifE and NifN from *K. oxytoca* (33). Nevertheless, intact NifEN from *Synechococcus* sp. JA-2–3B’a, *Hydrogenobacter thermophilus*, and *G. metallireducens* (hereafter denoted s *Sc*NifEN*^Ss^*, *Sc*NifEN*^Ht^,* and *Sc*NifEN*^Gm^*) were consistently detected in soluble protein extracts when produced and targeted to *S. cerevisiae* mitochondria (Fig. 3; Fig. S4). Thus, of 17 phylogenetically diverse NifEN scaffolds selected for analysis in the present work, three can be expressed in *S. cerevisiae* and targeted to the mitochondria in soluble forms, and all three can support *in vivo* FeMo-co biosynthesis when heterologously produced in *A. vinelandii*.

### Analysis of NifEN protein structures

Primary structure comparisons among the diverse NifEN variants indicated that those that were functional when expressed in *A. vinelandii* exhibited high identity when compared with *A. vinelandii* NifEN, ~40% identity for NifE and ~35% identity for NifN (Fig. S5A). An exception was the heterologously produced *K. oxytoca* NifEN which could not functionally substitute for *A. vinelandii* NifEN despite having high sequence identity when the primary structures are compared. NifEN variant structures, as predicted by AlphaFold (34, 35), were also compared with the crystallographically determined *A. vinelandii* NifEN structure (28). Those that could functionally replace *A. vinelandii* NifEN *in vivo* exhibited structures having the highest similarity to the *A. vinelandii* NifN (*P* = 0.008) (Fig. S5B). The level of similarity to NifN among the functional NifEN variants also correlated strongly with *in vivo* nitrogenase activity (*P* < 0.0001, Fig. S5C). No such correlation was found when the NifE structures were compared (*P* = 0.235) (Fig. S5D). Structural constraints imposed on NifE are likely to be more stringently conserved because it provides the site for NifB-co binding and FeMo-co completion (28). It is therefore likely that structural variability among NifN proteins is an important feature that affects NifEN functional compatibility with certain other components of *A. vinelandii* directing FeMo-co formation. Considering that NifEN interacts with several other proteins, such as NifB, NifX, NifV, NifQ, NifH, and NafY, further research could identify the structural features that are important for these interactions.

### Biochemical features of the selected NifEN variants produced in *A. vinelandii*

**Fig 4:**
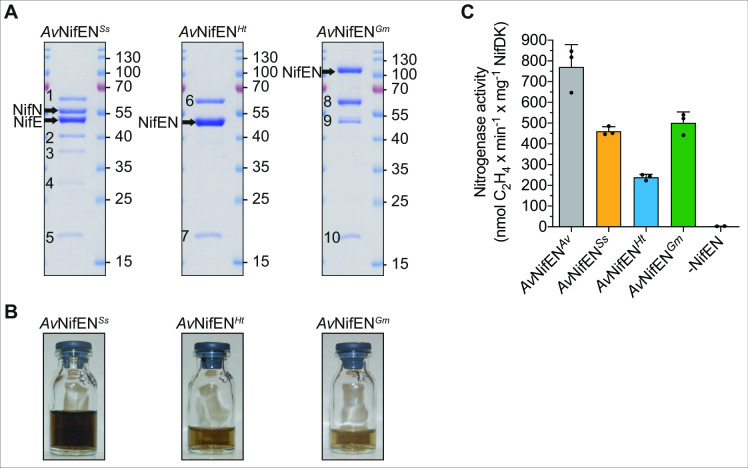
Isolation of NifEN variants from *A. vinelandii*. (**A**) Coomassie staining of *Av*NifEN*^Ss^*, *Av*NifEN*^Ht^*, and *Av*NifEN*^Gm^* isolated by STAC. Polypeptides numbered 1–10 were identified by peptide mass fingerprinting [1, 6, 8, and 9, pyruvate carboxylase subunit B (PycB, UniProt C1DH60); 2, 3, and 4, degradation products of *Av*NifN*^Ss^*; and 5, 7, and 10, biotin carboxyl carrier protein of acetyl-CoA carboxylase (AccB, UniProt C1DLJ8)]. Intact *Av*NifE*^Ht^* and *Av*NifN*^Ht^* migrate as a single band due to their similar size. Molecular weight markers (kDa) are indicated for each panel. (**B**) Appearance of *Av*NifEN*^Ss^*, *Av*NifEN*^Ht^*, and *Av*NifEN*^Gm^* isolated from *A. vinelandii*. Note that the differences in color between the samples may reflect differences in protein concentration rather than cluster occupancy/composition. (**C**) NifEN-dependent FeMo-co synthesis and apo-NifDK activation using *Av*NifEN*^Ss^*, *Av*NifEN*^Ht^*, and *Av*NifEN*^Gm^* isolated from *A. vinelandii* (see Materials and Methods for details). Units of activity are nanomoles of ethylene produced per minute and milligram of reconstituted NifDK. Error bars indicate mean ± SD; *n* = 3 technical replicates (*n* = 2 for the -NifEN control reaction) shown by dots.

Based on the results of the experiments used to identify soluble NifEN variants when expressed in *S. cerevisiae* and targeted to the mitochondria, the three soluble NifEN protein variants were isolated from *A. vinelandii* (denoted as *Av*NifEN*^Ss^*, *Av*NifEN*^Ht^*, and *Av*NifEN*^Gm^* for *Synechococcus* sp. JA-2–3B’a, *H. thermophilus*, and *G. metallireducens*, respectively) and tested for their ability to support *in vitro* FeMo-co formation (Fig. 4A). For this purpose, *Av*NifEN*^Ss^*, *Av*NifEN*^Ht^*, and *Av*NifEN*^Gm^* were hyper-expressed in an *A. vinelandii* strain that produces neither NifH nor NifDK as previously described (36, 37) for elevated expression of endogenous *A. vinelandii* NifEN (Table S4). Because NifH is required for processing NifB-co once it is bound to NifEN (13), the various NifEN scaffold proteins were expected to accumulate NifB-co rather than FeMo-co (Fig. 1) (29, 38). The identity of purified proteins was confirmed by mass spectrometry of samples displayed by SDS-PAGE (see legend to Fig. 4A). The presence of degradation products in the purified *Av*NifEN*^Ss^* protein is likely to reduce the specific activity of this protein variant due to complex with no or reduced functionality. Two common contaminants in STAC purifications from *A. vinelandii* cell extracts (PycB and AccB) were also detected in the eluates of the three NifEN variants. Accounting for the two permanent [4Fe-4S]-clusters and two [8Fe-9S-C]-containing NifB-co, the ideal Fe content of a NifEN heterotetramer fully loaded with NifB-co is 24 (28). The Fe content of isolated NifEN samples prepared in the present work ranged from 21 to 28 Fe atoms per NifEN protein, indicating that all of them are loaded with a significant fraction of NifB-co (Fig. 4B; Table S5). The ability of NifB-co-loaded *Av*NifEN*^Ss^*, *Av*NifEN*^Ht^*, and *Av*NifEN*^Gm^* to support FeMo-co formation was demonstrated by combining isolated NifB-co-loaded NifEN samples with NifH, homocitrate, and Mo to activate apo-NifDK (Fig. 4C) (13).

### Identification of optimum NifH and NifEN partners required for *in vitro* FeMo-co synthesis

**Fig 5:**
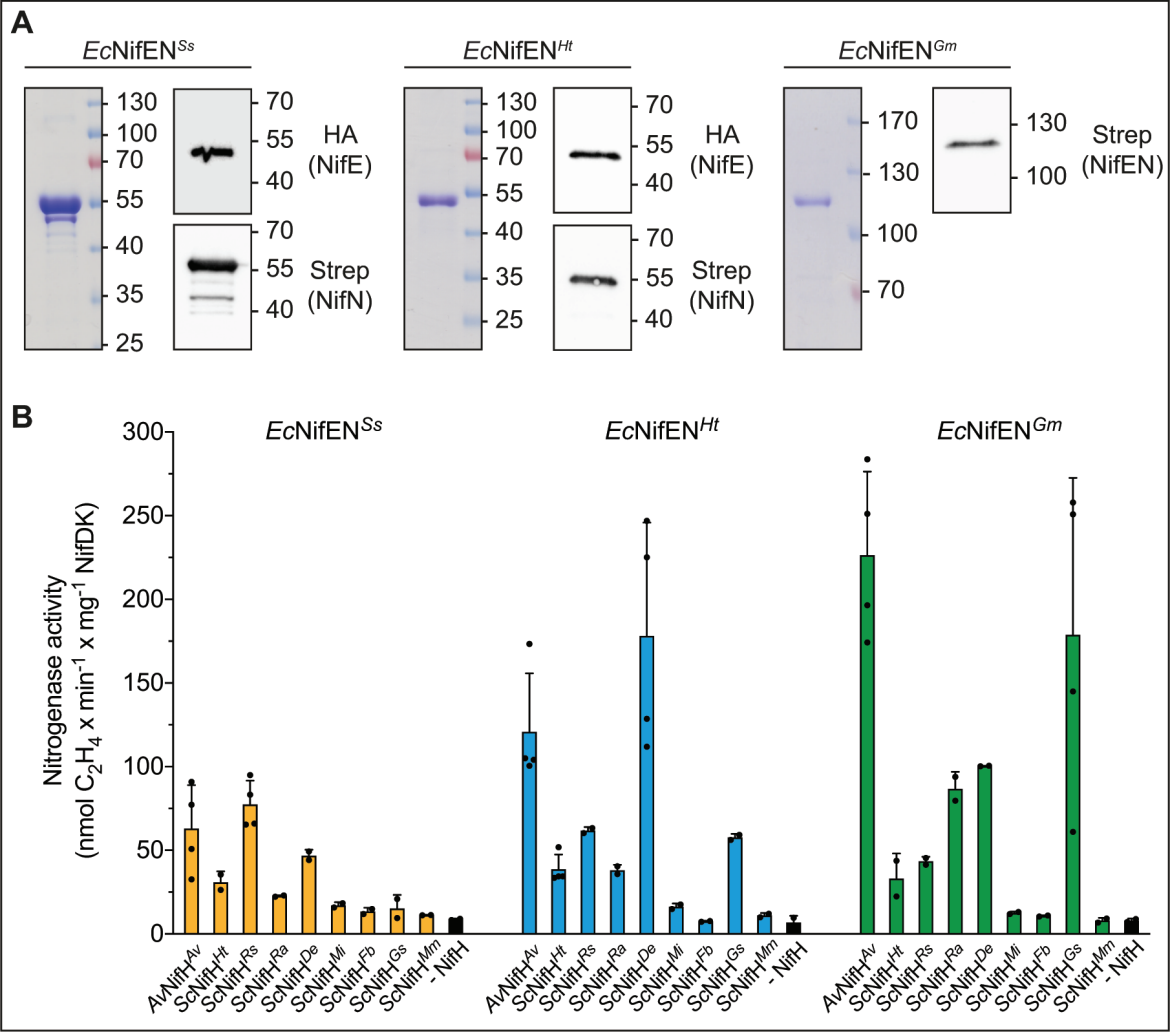
Compatibility of NifEN candidates with distinct NifH variants. (**A**) Coomassie staining and immunoblot analysis of *Ec*NifEN*^Ss^*, *Ec*NifEN*^Ht^*, and *Ec*NifEN*^Gm^* isolated by STAC from *E. coli*. Note that *Ec*NifE*^Ht^* and *Ec*NifN*^Ht^* migrate as a single band due to their similar size. The complete purification procedure is shown in Fig. S6. Molecular weight markers (kDa) and antibodies used are indicated. (**B**) NifB-co-dependent FeMo-co synthesis and apo-NifDK activation using *Ec*NifEN*^Ss^*, *Ec*NifEN*^Ht^*, or *Ec*NifEN*^Gm^* isolated from *E. coli* in combination with different *Sc*NifH*^Xx^* variants isolated from yeast. NifH*^Av^* isolated from *A. vinelandii* (*Av*NifH*^Av^*) was used as a control for acetylene reduction to eliminate the effect of different compatibilities between NifH variants and NifDK*^Av^*. Units of activity are nanomoles of ethylene produced per minute and milligram of reconstituted NifDK. The activity using apo-NifEN*^Av^* and NifH*^Av^* isolated from *A. vinelandii* strains DJ1118 and DJ, respectively, was 480 ± 110 units. Error bars indicate mean ± SD; *n* ≥ 2 technical replicates, shown by dots. *Av*, *A. vinelandii*; *Ht*, *H. thermophilus*; *Rs*, *Roseiflexus* sp.; *Ra*, *R. albus*; *De*, *D. ethenogenes*; *Mi*, *M. infernus*; *Fb*, *Firmicutes bacterium*; *Gs*, *G. sulfurreducens*; *Mm*, *M. marburgensis*.

NifH, homocitrate, and Mo are required for processing NifB-co on the NifEN scaffold to yield FeMo-co (Fig. 1). Thus, to accomplish effective processing of NifB-co by the soluble NifEN complexes, it was important to establish their functionality when combined with a NifH partner. *Av*NifEN*^Ss^*, *Av*NifEN*^Ht^*, and *Av*NifEN*^Gm^* have proven compatibility with *Av*NifH*^Av^*, as shown in the previous section. However, based on the work of Paya-Tormo and colleagues (20), other variants of NifH were found to be more soluble and therefore likely to be better suited for FeMo-co biosynthesis engineering. Using the same approach as described here, that study identified eight phylogenetically diverse NifH variants (denoted here as *Sc*NifH*^Xx^*) that accumulated as soluble proteins at high levels in the mitochondria of *S. cerevisiae* (20). Therefore, *in vitro* FeMo-co assembly assays were used to evaluate which of those NifH sources are functionally compatible when combined with NifEN*^Ss^*, NifEN*^Ht^*, and NifEN*^Gm^* for FeMo-co biosynthesis. Owing to the low yields of *Sc*NifEN*^Xx^* (0.4 mg, 0.9 mg, and 0.7 mg of *Sc*NifEN*^Ss^*, *Sc*NifEN*^Ht^*, and *Sc*NifEN*^Gm^* per 100 g yeast cells, respectively), NifEN species containing the two 4Fe-4S permanent clusters were heterologously produced in an *E. coli* strain expressing NifU*^Av^* and NifS*^Av^* (refer to Table S6 and the methods section in SI for details) and purified to survey their ability to direct FeMo-co formation when combined with various NifH sources produced in *S. cerevisiae* (Fig. 5A; Fig. S6). *E. coli* was also selected as a host for heterologous expression of the different NifEN forms to ensure they would not contain any endogenous NifB-co because *E. coli* does not encode a NifB. The three NifEN variants (denoted as *Ec*NifEN*^Ss^*, *Ec*NifEN*^Ht^*, and *Ec*NifEN*^Gm^*) were isolated from *E. coli* at sufficient amounts to perform *in vitro* FeMo-co synthesis assays. The iron content was lower than when isolated from *A. vinelandii* (Table S7), consistent with harboring only the 4Fe-4S permanent clusters. *In vitro* FeMo-co synthesis was performed from the precursor NifB-co (bound to NifX*^Av^*), molybdate, and homocitrate by combining distinct *Sc*NifH*^Xx^* variants with *Ec*NifEN*^Ss^*, *Ec*NifEN*^Ht^*, or *Ec*NifEN*^Gm^*. FeMo-co formation was evaluated by the ability of the assembly cocktail to activate apo-NifDK. The result of this survey revealed considerable variability in the capacity of distinct NifH variants to support NifEN-directed FeMo-co biosynthesis depending on the source of NifEN. Maximum activities were obtained when *Roseiflexus* sp. *Sc*NifH*^Rs^* was combined with *Ec*NifEN*^Ss^*, *Dehalococcoides ethenogenes Sc*NifH*^De^* with *Ec*NifEN*^Ht^*, and *Geobacter sulfurreducens Sc*NifH*^Gs^* with *Ec*NifEN*^Gm^*. Importantly, the maximum FeMo-co assembly activities obtained using *Ec*NifEN*^Ht^* and *Ec*NifEN*^Gm^* approached the optimized activities when NifEN and NifH isolated from *A. vinelandii* were combined to direct *in vitro* FeMo-co assembly (Fig. 5B).

### *In vitro* recapitulation of the FeMo-co biosynthetic pathway using only components produced in *S. cerevisiae*

**Fig 6:**
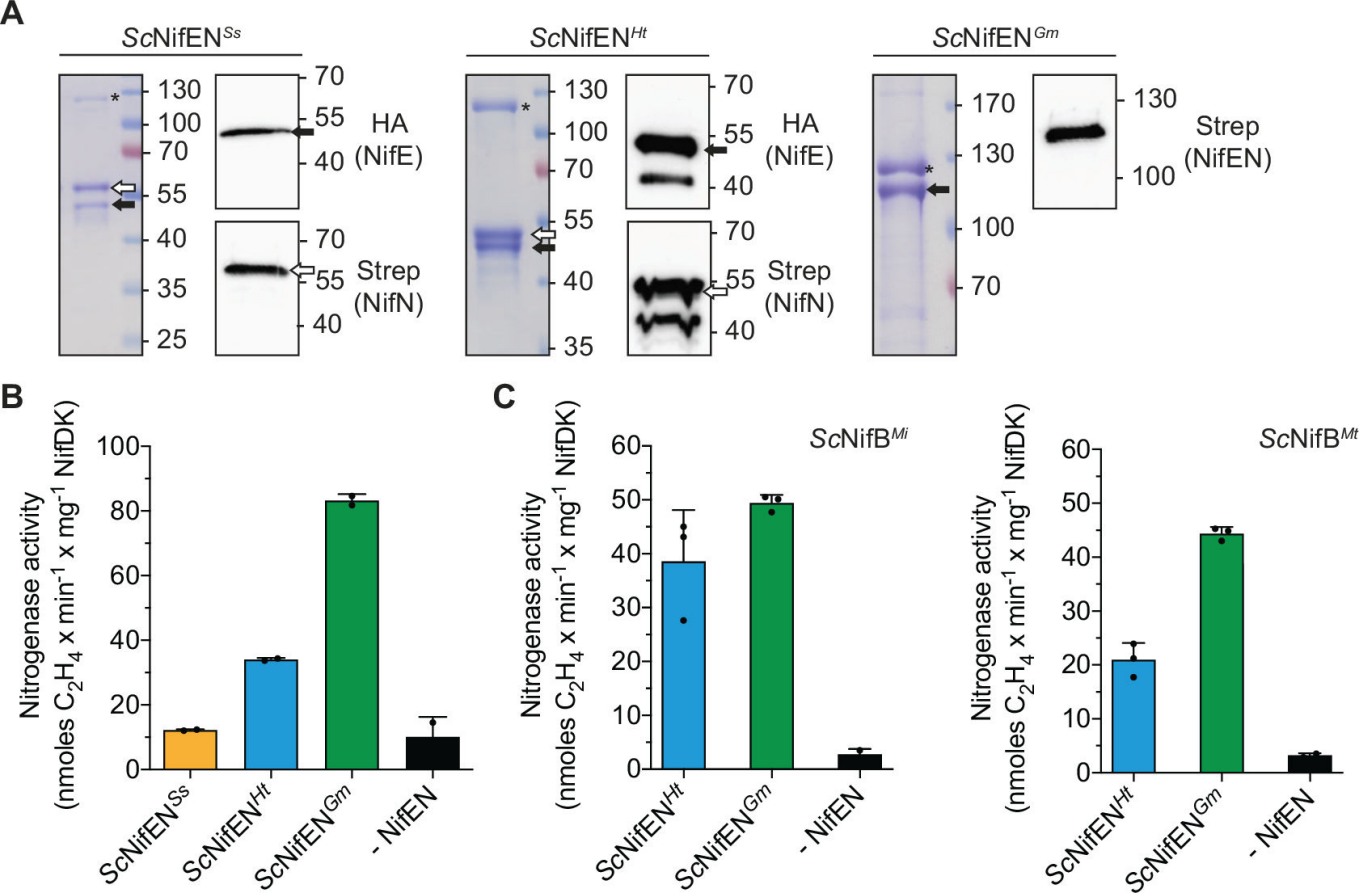
*In vitro* FeMo-co synthesis using NifEN isolated from *S. cerevisiae*. (**A**) Coomassie staining and immunoblot analysis of *Sc*NifEN*^Ss^*, *Sc*NifEN*^Ht^*, and *Sc*NifEN*^Gm^* isolated from yeast by STAC. The extra bands above the 40-kDa marker in *Sc*NifEN*^Ht^* samples might correspond to a NifEN degradation product. The complete purification procedure is shown Fig. S7. The black arrows point to NifE or NifEN fusion and white arrows to NifN. The black asterisks indicate protein often found in STAC purifications using *S. cerevisiae* extracts in our laboratory (previously identified as pyruvate carboxylase, N1P377). Molecular weight markers (kDa) and antibodies used are indicated. (**B**) NifB-co-dependent FeMo-co synthesis and apo-NifDK activation using NifEN and NifH proteins isolated from yeast (*Sc*NifEN*^Ss^* was combined with *Sc*NifH*^Rs^*, *Sc*NifEN*^Ht^* with *Sc*NifH*^De^*, and *Sc*NifEN*^Gm^* with *Sc*NifH*^Gs^*). The activity using apo-NifEN*^Av^* and NifH*^Av^* isolated from *A. vinelandii* strains DJ1118 and DJ, respectively, was 380 ± 53 units. Error bars indicate mean ± SD; *n* = 2 biological replicates shown by dots (≥4 technical replicates each). (**C**) NifB-dependent FeMo-co synthesis and apo-NifDK activation using NifB, NifEN, and NifH proteins isolated from yeast (*Sc*NifEN*^Ht^* with *Sc*NifH*^De^* and *Sc*NifEN*^Gm^* with *Sc*NifH*^Gs^*). Two different NifB variants, *M. infernus* (*Sc*NifB*^Mi^*) and *M. thermautotrophicus* (*Sc*NifB*^Mt^*), were tested. The activity using apo-NifEN*^Av^* and NifH*^Av^* isolated from *A. vinelandii* strains DJ1118 and DJ, respectively, was 873 ± 124 units (using *Sc*NifB*^Mi^*) and 653 ± 91 units (using *Sc*NifB*^Mt^*), respectively. Error bars indicate mean ± SD; *n* = 3 technical replicates (*n* = 2 for -NifEN, shown by dots). All activities are represented as nanomoles of ethylene produced per minute and milligram of NifDK.

Once optimum NifH partners for NifEN*^Ss^*, NifEN*^Ht^*, or NifEN*^Gm^* were identified, their soluble forms were isolated from mitochondria of aerobically cultured *S. cerevisiae* (Fig. 6A; Fig. S7) and combined with the corresponding optimum NifH partner (Fig. 5B), also isolated from *S. cerevisiae*, and evaluated for their ability to sustain *in vitro* FeMo-co biosynthesis. Isolated NifX with NifB-co bound was used in these initial FeMo-co biosynthesis cocktails. Substantial apo-NifDK activation was observed for the *Sc*NifH*^Gs^* and *Sc*NifEN*^Gm^* combination, while the activation was lower for the *Sc*NifH*^De^* and *Sc*NifEN*^Ht^* pair (Fig. 6B). No activation above background was observed when *Sc*NifH*^Rs^* and *Sc*NifEN*^Ss^* were combined, presumably indicating poor [4Fe-4S] cluster insertion and/or stability at this NifEN variant in the mitochondria. Therefore, we decided to exclude *Sc*NifEN*^Ss^* and perform FeMo-co biosynthesis only with the active proteins *Sc*NifEN*^Ht^* and *Sc*NifEN*^Gm^*. FeMo-co biosynthesis was finally evaluated using active forms of NifB, NifH, and NifEN proteins all isolated from *S. cerevisiae* mitochondria. For these experiments, two distinct NifB sources, *Methanocaldococcus infernus* (*Sc*NifB*^Mi^*) and *Methanothermobacter thermautotrophicus* (*Sc*NifB*^Mt^*), both of which can be produced in active forms in *S. cerevisiae* mitochondria, were used (15). Both *Sc*NifEN*^Ht^* and *Sc*NifEN*^Gm^* could activate apo-NifDK when combined with their corresponding optimum NifH partner and either of the two NifB variants (Fig. 6C). Therefore, the entire *in vitro* FeMo-co biosynthetic pathway can be recapitulated using only purified components produced in *S. cerevisiae*.

## DISCUSSION

Developing crop plants that can use atmospheric nitrogen by expressing genetic determinants for biological nitrogen fixation presents one of the most pressing challenges in modern plant engineering. Significant progress in synthetic biology applications, the protection of O_2_-sensitive molecules by organellar targeting, and the identification of the minimum gene set needed to produce an active nitrogenase has fostered hope of engineering nitrogen-fixing plants (8). One of the primary obstacles is the assembly of the nitrogenase active-site metallocluster, called FeMo-co, which demands the coordinated action of at least NifU, NifS, NifH, NifV, NifQ, NifB, NifE, and NifN, in a sequential and multi-step process (13). This study uses NifH, NifB, and NifEN proteins that were produced, targeted, and extracted from the mitochondria of *S. cerevisiae* to synthesize FeMo-co *in vitro*. This achievement builds upon previous studies that reported key steps in FeMo-co biosynthesis and nitrogenase formation in model eukaryotes. These include the expression of active NifH, the most O_2_-sensitive catalytic component of nitrogenase, which is also involved in FeMo-co biosynthesis (19, 20, 25), and the *in vivo* production of NifB-co, a precursor to FeMo-co that acts as sole source of both Fe and S (15). The formation of the other catalytic component of nitrogenase, NifDK, has also been shown, although in a form not readily activated by FeMo-co (18). These milestones represent defining proofs of principle that it may be possible to complete the biosynthetic pathway for functional nitrogenase formation in the mitochondria of yeast. Furthermore, synthetic biology techniques have effectively facilitated the expression of all genes required for nitrogen fixation in model eukaryotes (18, 39). Nonetheless, these efforts have yet to produce a nitrogen-fixing eukaryote. For this, it is crucial that the Nif proteins expressed in eukaryotes retain their catalytic activities and that they can interact with each other to establish an efficient nitrogenase biosynthetic pathway.

Two potential approaches may be employed to resolve this issue: firstly, to investigate why a specific element essential for nitrogen fixation cannot be expressed heterologously in an active manner and then engineer it to be better expressed in the new host and secondly, to survey nitrogen fixation components from the phylogenetic diversity to identify those that can be functionally produced in the eukaryote host without the need for further modification. While the second approach, utilized in the present study and previous ones (20, 21, 25), is straightforward, it brings about technical challenges. Specifically, a group of interacting components is necessary to synthesize an operational nitrogenase, implying that an expressed protein demonstrating individual activity in a eukaryote must possess the capability to engage constructively with other components. This is especially relevant for the biosynthesis of FeMo-co, which involves a pathway of interdependent assembly scaffolds as shown in Fig. 1. Hence, each assembly scaffold must undergo experimental validation when combined with the other proteins involved in the formation and insertion of FeMo-co into nitrogenase. For this, cluster-transfer proteins such as NifX and NafY that facilitate the delivery of NifB-co and FeMo-co between assembly scaffolds can introduce some flexibility in pathway engineering (13).

This study replicates the FeMo-co biosynthesis pathway by combining assembly components produced in *S. cerevisiae* mitochondria. This approach can benefit from utilizing nitrogenase components that are phylogenetically distinct and possess properties that make them better suited for use in plant cells. For example, NifH from *G. sulfurreducens* has been shown to have an increased tolerance toward O_2_ (20), and NifH from *D. ethenogenes* does not necessitate NifM for maturation, resulting in less genes to transfer into the plant genome (20). In this regard, the expression of a natural NifEN fusion, such as the one found in *G. metallireducens*, not only reduces the genetic elements required but also ensures the correct stoichiometric ratio between the two gene products.

It is worth mentioning that the *in vitro* FeMo-co biosynthesis assay, which utilizes phylogenetically diverse proteins isolated from *S. cerevisiae*, is dependent on the activation of apo-NifDK derived from *A. vinelandii*. It is not currently feasible to determine FeMo-co assembly and nitrogenase activation *in vivo* because a FeMo-co-activatable form of apo-NifDK has yet to be produced in a eukaryotic host. However, this subject is under intense investigation in several research laboratories using a range of different approaches. For example, this study implements the phylogenetic survey approach, while others have employed translational fusions (27) or a posttranslational protein splicing strategy, which may offer particular benefits for complex biosynthetic pathways that require careful balance of component expression (40). The implementation of these methods has the potential to significantly decrease the quantity of transcriptional units required and thus simplify the transformation of plant genomes. Furthermore, they aid in organelle targeting and ensure correct stoichiometry of protein complexes, crucial for some proteins such as NifEN or NifDK (40, 41). However, it is important to ascertain the impact of engineering such vast polypeptides on expression, mitochondrial physiology, and functionality. Although both natural NifEN fusions from *A. variabilis* and *G. metallireducens* tested in this study were shown to be functional *in vivo* inside *A. vinelandii* (Fig. 2), the nitrogen fixing activities of both strains were relatively low. This outcome suggests poor NifEN expression and/or stability. Once a suitable NifDK protein for expression in a model eukaryote, such as *S. cerevisiae*, has been identified—whether natural or engineered—the FeMo-co biosynthetic modules presented in this study could be used to produce a functional nitrogenase *in vivo*. Such a breakthrough would pave the way for the first nitrogen-fixing eukaryotic cell.

Results reported here should also have practical merit in further elucidation of details concerning FeMo-co assembly on the NifEN scaffold. Thorough investigations about NifEN in *A. vinelandii* and *E. coli* have provided significant insights into NifEN expression and functionality (13). It is recognized that this scaffold serves as the site for both Mo insertion and homocitrate attachment to NifB-co; however, the specific mechanisms involved in this process remain unknown. The availability of a heat stable and functional form of NifEN that is naturally a NifEN fusion protein offers new experimental tools to explore the order and mechanism of specificity for Mo insertion and homocitrate attachment to NifB-co.

## MATERIALS AND METHODS

### Expression and isolation of recombinant NifEN proteins from *A. vinelandii*

For expression and isolation of NifEN from large‐scale *A. vinelandii* cultures, cells were grown in a 150-L custom‐built fermenter (W.B. Moore Inc., Easton, PA) at 30°C in a modified Burk’s medium containing 5.7 mM NH_4_AOc as the nitrogen source. Cells were grown until exhaustion of fixed nitrogen and derepressed for 3 h more prior harvesting.

The Strep‐tagged NifEN proteins were purified from the *A. vinelandii* cell paste following previously described procedures using Strep‐Tactin columns (IBA Lifesciences) (42), with some modifications. Briefly, around 100 g of cells was resuspended in anaerobic buffer A [50 mM Tris-HCl, 2 mM sodium dithionite (DTH), and pH 8]. The resuspended cells were lysed using Nano-DeBee homogenizer maintaining a stream of Ar over the reservoir during the disruption process. Disrupted cells were supplemented with 1 mL of 100 mM phenylmethylsulfonyl fluoride (PMSF), 10 mg of DNAse I, and 5 mg of Pepstatin A. Cell extract was centrifuged at 167,000 × *g* for 45 min at 4°C (Type 45 Ti, Beckman Coulter). The cell-free extracts were filtered and loaded at 2 mL/min into a 5-ml Strep-Tactin column (IBA Lifesciences). The column was washed with buffer B (50 mM Tris-HCl, 500 mM NaCl, 2 mM DTH, and pH 8.0). Protein bound to the column was eluted with buffer B supplemented with 50 mM biotin. Finally, purified NifEN proteins were snap frozen and stored in liquid N_2_ for further analysis.

### Expression and isolation of NifEN from *E. coli*

Protein expression in *E. coli* BL21-DE3 was performed in 3-L flasks containing 1 L of LB supplemented with 20 µM ammonium iron (III) citrate and antibiotics in the growth phase and 3 g/l lactose, 2 mM L-cysteine, and 0.2 mM ammonium iron (III) citrate in the expression phase (43). Expression of proteins was performed overnight at 30°C and 105 rpm. The cells were collected by centrifugation at 5,000 × *g* for 5 min at 4°C, frozen in liquid N_2_, and stored at −80°C.

The NifEN protein purification procedures were performed in anaerobic chambers at <1  ppm O_2_, containing 5% of hydrogen and 95% of nitrogen (Coy Laboratories). Cells were resuspended in anaerobic buffer A (100 mM Tris-HCl, 200 mM NaCl, 10% glycerol, 2 mM DTH, and pH 8) supplemented with 1 mM PMSF, 1 µg/mL leupeptin, and 5 µg/mL DNAse I at a ratio 1:2 (wt/vol). The resuspended cells were lysed using an EmulsiFlex-C5 homogenizer (Avestin Inc.) operating at 15,000 psi. Broken cell extract was transferred to centrifuge tubes equipped with sealing closures (Beckman Coulter) and centrifuged at 50,000 × *g* for 1 h at 4°C (Avanti J-26 XP, Beckman Coulter). The supernatant containing soluble proteins was loaded at 2 mL/min into a 5-mL Strep-Tactin XP column (IBA LifeSciences) attached to an ÄKTA FPLC (GE Healthcare). Then, the column was washed with 100 mL of anaerobic buffer A, and bound NifEN was eluted using 20 mL anaerobic buffer A supplemented with 50 mM biotin. Isolated NifEN proteins were concentrated using Amicon Ultra-15 centrifugal filters with a 100-kDa cut-off (Millipore). The remaining biotin present in the sample was removed using PD-10 desalting columns (GE Healthcare) according to the manufacturer’s instructions. Finally, purified NifEN proteins were snap frozen and stored in cryovials (Nalgene) under liquid N_2_.

### Expression and isolation of NifEN from *S. cerevisiae*

For expression of *Sc*NifEN*^Ss^ Sc*NifEN*^Ht^* or *Sc*NifEN*^Gm^* together with SU9-NifU*^Av^* and SU9-NifS*^Av^*, *S. cerevisiae* was grown in 4-L fermenters (Applikon, VERTEX Technics) under aerobic conditions (0.625 L of air per minute and liter of culture) and 250 rpm stirring as previously described (15). A typical fermenter procedure (Fig. S8) generated 200–220 g of cells that were stored in liquid N_2_.

STAC purifications of yeast-expressed NifEN variants were performed as described above for NifEN produced in *E. coli*, with the following modifications. Two hundred grams of frozen yeast pellets was resuspended in 400 mL of anaerobic buffer B (100  mM Tris-HCl, 300  mM NaCl, 10% glycerol, 2  mM DTH, and pH 8.5) supplemented with 1 mM PMSF, 1 µg/mL leupeptin, 5 µg/mL DNAse I, and 1:200 (vol/vol) BioLock (IBA Lifesciences). The cells were lysed using a HPH 2000/4-DH5 high-pressure homogenizer (IKA) operating at 20,000 psi under anaerobic conditions. The soluble protein extracts were additionally filtered by passing through a 0.2 µm pore-size filter (Nalgene Rapid-Flow, Thermo Scientific) before loading into the column. Sample loading, column washing, protein elution, NifEN concentration, buffer exchange (desalting), and protein storage were identical as in the above-described procedure.

### *In vitro* FeMo-co synthesis and apo-NifDK activation assays

FeMo-co synthesis was determined *in vitro* by measuring the amount of apo-NifDK activated by FeMo-co using acetylene reduction assays. This process can be divided into four separate steps: the first three corresponding to FeMo-co synthesis and insertion into apo-NifDK (Fig. 1) and the fourth being the determination of reconstituted NifDK activity. Step 1 is synthesis and transfer of NifB-co to NifEN. Step 2 is conversion of NifB-co to FeMo-co at NifEN in a process that incorporates Mo and homocitrate and that, in addition to NifEN, requires the action of NifH. Step 3 is transfer of FeMo-co from NifEN to apo-NifDK (P-cluster containing but FeMo-co-deficient NifDK) to form active NifDK. Step 4 is reduction of acetylene into ethylene at NifDK in the presence of excess NifH and with DTH as reductant.

All steps were performed inside anaerobic chambers (Coy Laboratories). *In vitro* FeMo-co synthesis and insertion into apo-NifDK (steps 1–3) were performed in 100-µL reactions in Eppendorf tubes at 30°C with shaking at 600 rpm for 60 min in reaction mixtures with distinct composition depending on the type of assay, as specified in the following specific sections, and ATP regenerating mixture [1.23 mM ATP, 18 mM phosphocreatine disodium salt, 2.2 mM MgCl_2_, 3 mM DTH, and 40 µg/mL creatine phosphokinase in 100 mM MOPS (pH 7.0)]. After the FeMo-co synthesis reaction, 17.5  µM (NH_4_)_2_MoS_4_ was added and incubated for 30 min at 30°C to inhibit insertion of FeMo-co that could be synthesized by NifEN*^Xx^* and NifH*^Av^* during the acetylene reduction assays (44). For the determination of reconstituted NifDK (step 4), the FeMo-co synthesis and insertion mixture was transferred to a 9 mL glass vial containing 500 µl of ATP regenerating mixture to which 1.0 µM NifH*^Av^* was added (corresponding to a final molar ratio of NifH:NifDK of 20:1). Then, the N_2_ atmosphere in the headspace was exchanged for Ar and 0.75 mL acetylene was injected. The vials were incubated with shaking in a water bath for 20 min at 30°C. Finally, acetylene reduction activity was stopped by the addition of 100 µL of 8 M NaOH. Produced ethylene was measured by injecting 50 µL of the gas phase into a gas chromatograph (GC-2014, Shimadzu) equipped with a Porapak N 80/100 column.

*In vitro* FeMo-co synthesis assays can be initiated at step 1 in two different ways (13): the NifB-dependent assay that uses NifB together with a source of [4Fe-4S] clusters (either delivered by NifU or directly assembled on NifB using Fe and S) or the NifB-co-dependent assay that uses NifB-co directly (usually isolated from a diazotrophic bacterium in complex with the NifX carrier protein). The *in vitro* FeMo-co synthesis assay can also be initiated at step 2 using NifEN containing the FeMo-co precursor derived from NifB-co. For simplicity, this reaction is herein called NifEN dependent.

For *in vitro* FeMo-co synthesis and apo-NifDK activation using NifEN variants isolated from *A. vinelandii* (Fig. 4C), the NifEN-dependent assay was used. The 100 µL FeMo-co synthesis reaction mixture contained 17.5  µM Na_2_MoO_4_, 175  µM *R*-homocitrate, 0.6 µM *Av*NifEN*^Xx^*, 3.0 µM NifH*^Av^*, 0.3 µM apo-NifDK*^Av^*, 1 mg/mL BSA, and ATP-regenerating mixture. Positive control reactions contained 0.6 µM NifB-co-loaded NifEN*^Av^* isolated from *A. vinelandii* strain DJ1041 (*ΔnifHDKTYorf1orf2 his-nifEN*) (29), and for negative control reactions no NifEN was added.

For *in vitro* FeMo-co synthesis and apo-NifDK activation using *Ec*NifEN*^Xx^* variants isolated from *E. coli* (Fig. 5B), step 1 was performed with NifB-co bound to GST-tagged NifX, produced *in vivo*, and isolated from a *K. oxytoca* strain lacking NifEN (UC32) as previously described (45). To compare the different NifEN activities, NifEN protein was added based on Fe concentration instead of protein concentration (as the isolated *Ec*NifEN*^Xx^* proteins had incomplete Fe-S cluster occupancy). The 100 µL FeMo-co synthesis reaction mixture contained GST-tagged NifX-NifB-co (9.6 µM Fe), 17.5  µM Na_2_MoO_4_, 175  µM *R*-homocitrate, *Ec*NifEN*^Xx^* (4.8 µM Fe), 3.0 µM *Sc*NifH*^Xx^*, 0.3 µM apo-NifDK*^Av^*, 1  mg/mL BSA, and ATP-regenerating mixture. Positive control reactions contained 0.6 µM apo-NifEN*^Av^* isolated from *A. vinelandii* strain DJ1118 (*ΔnifB::kan^R^ PnifH::polyHis::nifEN*), which corresponds to the *A. vinelandii* strain DJ1041 (expresses His-tagged NifEN) (29) with a deleted *nifB* gene. For negative control reactions no NifEN was added.

For *in vitro* FeMo-co synthesis and apo-NifDK activation using *Sc*NifEN*^Xx^* isolated from *S. cerevisiae* (Fig. 6B and C), step 1 was performed with either isolated NifB-co bound to the cluster-transfer protein NifX (Fig. 6B) as described above or with *Sc*NifB*^Xx^* variants (Fig. 6C) isolated from *S. cerevisiae* co-expressing NifU, NifS, and FdxN as previously described (15). For the NifB-co-dependent FeMo-co synthesis assays (Fig. 6B), the 100 µL FeMo-co synthesis reaction mixture contained GST-tagged NifX-NifB-co (9.6 µM Fe), 17.5 µM Na_2_MoO_4_, 175 µM *R*-homocitrate, 0.6 µM *Sc*NifEN*^Xx^*, 3.0 µM *Sc*NifH*^Xx^*, 0.3 µM apo-NifDK*^Av^*, 1 mg/mL BSA, and ATP-regenerating mixture. For the NifB-dependent FeMo-co synthesis assays (Fig. 6C), the 100-µL FeMo-co synthesis reaction mixture contained 1.2 µM *Sc*NifB*^Xx^* isolated from *S. cerevisiae* (15), 10.8 µM [4Fe-4S] cluster from NifU isolated from *E. coli* and reconstituted using NifS, Fe and cysteine as previously described (21), 125 µM SAM, 1.2 µM NifX isolated from *E. coli*, 17.5 µM Na_2_MoO_4_, 175 µM *R*-homocitrate, 0.6 µM *Sc*NifEN*^Xx^*, 3.0 µM *Sc*NifH*^Xx^*, 0.3 µM apo-NifDK*^Av^*, 1 mg/mL BSA, and ATP-regenerating mixture. Positive control reactions for both assays contained 0.6 µM apo-NifEN*^Av^* isolated from *A. vinelandii* strain DJ1118 (*ΔnifB::kan^R^ PnifH::polyHis::nifEN)*, and for negative control reactions, no NifEN was added.

## Data Availability

Accession numbers of genes used in this manuscript are listed in Table S1. Additional methods are described in Supplementary Material.

## SUPPLEMENTAL MATERIAL

Supplemental Information. mbio.03088-23-s0001.pdf Supplemental Materials and Methods, Figures S1 to S8, and captions to Tables S1 to S7.

Table S1 mbio.03088-23-s0002.xlsx Metabolism and growth conditions for the organisms from which the NifEN library candidates were selected, yeast-optimized DNA sequences, and the corresponding protein sequences.

Table S2 mbio.03088-23-s0003.xlsx DNA sequences used for Δ*nifEN* complementation in DJ2898, sequences of the expressed NifEN proteins, and the resulting *A. vinelandii* strains.

Table S3 mbio.03088-23-s0004.xlsx DNA sequences used for *nifEN* expression in *S. cerevisiae*, sequences of the expressed NifEN proteins, plasmids generated, and the resulting yeast strains.

Table S4 mbio.03088-23-s0005.xlsx DNA sequences used for heterologous *nifEN* expression in DJ, sequences of the expressed NifEN proteins, and the resulting *A. vinelandii* strains.

Table S5 mbio.03088-23-s0006.xlsx Fe atoms per NifEN molecule and yield of NifEN protein from *A. vinelandii* cells.

Table S6 mbio.03088-23-s0007.xlsx DNA sequences used for *nifEN* expression in *E. coli* and sequences of the expressed NifEN proteins.

Table S7 mbio.03088-23-s0008.xlsx Fe atom per purified NifEN protein and yield of NifEN from *E. coli* cells.

ASM does not own the copyrights to Supplemental Material that may be linked to, or accessed through, an article. The authors have granted ASM a non-exclusive, world-wide license to publish the Supplemental Material files. Please contact the corresponding author directly for reuse.
