# Supplementary material for: Nitrogenase cofactor biosynthesis using proteins produced in mitochondria of Saccharomyces cerevisiae
Source: mBio. 2023 Dec 21;15(2):e03088-23. doi: 10.1128/mbio.03088-23 (PMC10865832; doi:10.1128/mbio.03088-23)
Supplement: Supplemental Information. — Supplemental Materials and Methods, Figures S1 to S8, and captions to Tables S1 to S7. [file mbio.03088-23-s0001.pdf]

SUPPLEMENTARY INFORMATION

Nitrogenase Cofactor Biosynthesis using proteins produced in mitochondria of *Saccharomyces cerevisiae*

Katarzyna Dobrzyńska, Ana Pérez-González, Carlos Echavarri-Erasun, Diana Coroian, Alvaro Salinero-Lanzarote, Marcel Veldhuizen, Dennis R. Dean, Stefan Burén, Luis M. Rubio

CONTENTS

Supplementary Materials and Methods

Figure S1. Alignment of NifE and NifN proteins depicting conserved cluster-binding Cys residues.

Figure S2. *In vivo* functionality of the 17 NifEN candidates in *A. vinelandii*.

Figure S3. Expression of NifEN variants in *S. cerevisiae*.

Figure S4. Co-purification and purification experiments to identify soluble NifEN variants accumulating in the mitochondria of aerobically cultured *S. cerevisiae*.

Figure S5. Sequence and structural divergence analysis of NifEN proteins in the library.

Figure S6. STAC purification of NifEN variants expressed in *E. coli* cells.

Figure S7. STAC purification of NifEN variants expressed in aerobic cultures of *S. cerevisiae*.

Figure S8. Typical batch fermentation process of the *S. cerevisiae* strain expressing *Sc*NifEN^{Gm} (KD37Y).

Table S1. Metabolism and growth conditions for the organisms from which the NifEN library candidates were selected, yeast-optimized DNA sequences, and the corresponding protein sequences.

Table S2. DNA sequences used for *ΔnifEN* complementation in DJ2898, sequences of the expressed NifEN proteins, and the resulting *A. vinelandii* strains.

Table S3. DNA sequences used for *nifEN* expression in *S. cerevisiae*, sequences of the expressed NifEN proteins, plasmids generated, and the resulting yeast strains.

Table S4. DNA sequences used for heterologous *nifEN* expression in DJ, sequences of the expressed NifEN proteins, and the resulting *A. vinelandii* strains.

Table S5. Fe atoms per NifEN molecule and yield of NifEN protein from *A. vinelandii* cells.

Table S6. DNA sequences used for *nifEN* expression in *E. coli* and sequences of the expressed NifEN proteins.

Table S7. Fe atom per purified NifEN protein and yield of NifEN from *E. coli* cells.

SUPPLEMENTARY MATERIALS AND METHODS

Generation of the *nifEN* library. Gene sequences were optimized for expression in *S. cerevisiae* using the GenSmart Codon Optimization tool (GenScript) using protein sequences from UniProt (1). All DNA parts were synthesized by GenScript and cloned into pUC cloning vectors (Table S1). The *nifE* and *nifN* genes were synthesized without stop codon and were flanked by BamHI and BspEI restriction sites at the 5´ and 3´ ends, respectively (*i. e*., GGATCC**ATG**…TCCGGA).

Generation of plasmids and strains for NifEN protein expression in *A. vinelandii*. For *in vivo* complementation experiments in the *A. vinelandii* strain DJ2898 (*ΔvnfDGK, ΔanfHDGK:Gent*^{R}*, ΔnifEN:kan*^{R}*, str*^{R} in *scr* region), the 17 *nifEN* candidate gene cassettes were cloned into a plasmid suitable for *A. vinelandii* transformation. Two strategies were implemented to avoid improper *nifEN* gene translation. First, the native intergenic sequence naturally found between *nifE* and *nifN* in *A. vinelandii* was inserted between the yeast codon-optimized *nifE* and *nifN* variants using overlapping PCR, except for the two *nifEN* natively fused (from *A. variabilis* and *G. metallireducens*). In addition, the last three codons of *nifE* and the first three codons of *nifN* were replaced by the corresponding codons from the *A. vinelandii nifE* and *nifN* genes by PCR. The *nifEN* cartridges were then subcloned into plasmid pN2KD163 using exonuclease and ligation-independent cloning (ELIC) (2). Plasmid pN2KD163 is a derivative of pDB1588 that contains recombination sites for integrating the genes into the sucrose (*scr*) metabolism gene region of the *A. vinelandii* genome and under the arabinose promoter (*P*ara) regulation (3). A DNA sequence encoding an SS-tag was placed at the 5´ of each yeast-optimized *nifE*

gene to facilitate NifE protein detection and NifEN isolation. Strain DJ2898 was independently transformed with the 17 plasmids containing the *nifEN* candidate gene (Table S2).

For NifEN protein isolation from *A. vinelandii*, the yeast codon-optimized *nifEN* genes were cloned into NheI and NsiI digested pDB2617 (placing an SS-tag at the 5´-end of the *nifE* gene) that allows for recombination between the *nifH* promoter and *nifX* in *A. vinelandii.* The resulting plasmids were used to transform *A. vinelandii* DJ, replacing *nifHDKTYnafABnifEN* by the respective *nifEN* gene, following the procedure used to generate strain DJ1041 (4). The plasmids and the resulting strains used in this work are listed in Table S4.

***A. vinelandii* transformation and growth.** Genetic modifications were incorporated into the *A. vinelandii* genome by transformation of competent cells using previously described methodology (5). Gene deletions and insertions were confirmed by PCR and sequencing of the amplified genomic DNA (Genomics Sequencing Center, Fralin Life Sciences Institute, Virginia Tech). Transformed *A. vinelandii* cells were cultured for five days at 30°C on solid Burk's modified nitrogen-free medium (6) supplemented with 1 µM Na_2MoO_4 and 3 g/l arabinose (required for inducing the expression of the *nifEN* gene variants). For non-diazotrophic conditions, 13 mM NH_4AOc was added to the media as the nitrogen source.

***In vivo* acetylene reduction assays in *A. vinelandii*.** Fresh *A. vinelandii* biomass was used to inoculate 125 ml flasks containing 25 ml of Burk medium supplemented with 5 mM NH_4AOc at an initial OD_{600} of 0.00125. Flasks were cultured overnight at 30°C with constant agitation at 250 rpm. The next day, the

presence of fixed nitrogen source in the media was confirmed by Nessler reagent. Hundred twenty-five ml flasks containing 25 ml of Burk's medium without NH_4AOc but containing 3 g/l arabinose were inoculated at an initial OD_{600} of 0.3 and then incubated at 30°C and 250 rpm for several hours until cell cultures reached an OD_{600} of about 1. At this point, *in vivo* acetylene reduction assays were performed by transferring 1 ml of each culture to 9 ml glass vials fitted with butyl rubber caps. Five hundred µl of acetylene was injected and overpressure was relieved. The vials were incubated at 30°C for 15 min. The reactions were then stopped by injecting 100 µl of 8 M NaOH. The concentrations of ethylene in the headspace of the vials were measured using a gas chromatograph quipped with a flame ionization detector (GC-2010 Plus, Shimadzu).

Generation of plasmids and strains for NifEN expression in *E. coli*. To generate pN2KD140 (for expression of TS-NifEN from *G. metallireducens*), pN2KD142 (for expression of HA-NifE/NifN-SS from *Synechococcus* sp. JA-2-3B'a) and pN2KD143 (for expression of HA-NifE/NifN-SS from *H. thermophilus*), the genes and tag sequences were amplified by PCR from yeast expression vectors (Table S3) and inserted into NcoI+XhoI (for TS-NifEN), NcoI+BamHI (for HA-NifE) or NdeI+XhoI (for NifN-SS) digested pETDuet-1 vectors (Novagen) using ELIC, (Table S6).

Plasmids for expression of *nifEN* genes were introduced in *E. coli* strain BL21 (DE3) by transformation together with pN2LP30 (7), a plasmid for the inducible expression of *nifU, nifS,* and *metK*.

Generation of plasmids and strains for NifEN expression in *S. cerevisiae*. The pESC-HIS vector (Agilent Technologies) was used for NifE

expression. First, the BamHI site within pESC-HIS was replaced by NcoI using PCR, generating plasmid pN2KD13. Then, a fragment containing SU9-HA with a sequence for BamHI-BspEI-Stop added to the 3´-end of SU9-HA was synthesized by GenScript and inserted using ELIC into NotI+SacI digested pN2KD13, generating pN2KD64 (pESC-HIS with SU9-HA-BamHI-BspEI-Stop). The *nifE* genes were then inserted into BamHI+BspEI digested pN2KD64 by cloning, generating pESC-HIS variants for expression of SU9-HA-NifE.

The pESC-LEU vector (Agilent Technologies) was used for NifN expression. First, a fragment containing SU9-Strep tag II (denoted here as SS-tag) with a sequence for BamHI-BspEI recognition sites inserted between the SU9 mitochondria signal and the SS-tag, and a Stop sequence added to the 3´-end of SS-tag, was synthesized by GenScript, and inserted using ELIC into NotI+SacI digested pN2KD13, generating pN2KD40 (pESC-HIS with SU9-BamHI-BspEI-SS- Stop). Then, the XhoI+PacI fragment from pN2KD40 was inserted by cloning into XhoI+PacI digested pESC-LEU, generating pN2KD65 (pESC-LEU with SU9-BamHI-BspEI-SS-Stop). The *nifN* genes were then inserted into BamHI+BspEI digested pN2KD65 by cloning, generating pESC-LEU variants for expression of SU9-NifN-SS.

The pESC-HIS vector (Agilent Technologies) was used for NifEN fusion protein expression. First, the sequence encoding for SU9-Twin Strep tag (denoted here as TS-tag) was amplified by PCR from plasmid pN2SB103 (8) and inserted using ELIC into NotI+SacI digested pN2KD13, generating pN2KD66 (pESC-HIS with SU9-TS-BamHI-BspEI-Stop). The *nifEN* fusion genes were then inserted into

BamHI+BspEI digested pN2KD66 by cloning, generating pESC-HIS variants for expression of SU9-TS-tagged NifEN.

The DNA sequences used, and the constructed plasmids and strains, are listed in Table S3. All restriction enzymes were purchased from New England Biolabs. T4 DNA ligase (Promega) was used to insert the DNA fragments into the target vector. The constructed plasmids were introduced into chemically competent *E. coli* (strains DH5α or TOP10) and transformed cells were selected on solid LB medium supplemented with appropriate antibiotics. Plasmid isolation was performed using Qiaprep Spin Miniprep kit (Qiagen) and sequences were verified by Sanger sequencing (Macrogen). All yeast plasmids were introduced into parental strain SB320Y, which expressed NifU^{Av} and NifS^{Av} targeted to mitochondria following the lithium acetate transformation method (9). All yeast strains used in this work originated from *S. cerevisiae* W303–1A (ura3–1; trp1–1; leu2–3112; his3–11; ade2–1; can1–100).

Screening of NifEN solubility and complex formation in *S. cerevisiae.* Single yeast colonies were used to inoculate flasks containing 10 ml of synthetic drop-out (SD) medium supplemented with 2% glucose and appropriate amino acids (10). The precultures were incubated overnight at 28°C with shaking at 200 rpm. To induce NifEN, NifU and NifS protein expression, yeast cells were transferred to SD medium containing galactose instead of glucose, and supplemented with 0.1% peptone and 0.1% yeast extract, at an initial OD of 0.3, and cultured for an additional 20 h.

Total and soluble yeast protein extracts were prepared by centrifuging yeast cells at 5,000 x *g* for 10 min at 4°C, and then incubating yeast pellets for 20 minutes at room temperature in YeastBuster protein extraction reagent (Novagen) with shaking at 800 rpm in an anaerobic chamber (Coy Laboratories). Fifty µl of the resulting total extract was mixed with 2x Laemmli buffer and heated for 10 min at 95°C. To prepare soluble extract, the insoluble cell debris was removed by centrifuging the total extract at 16,000 x *g* for 20 min at 4°C. Fifty µl of the resulting supernatant was mixed with 2x Laemmli buffer and heated for 10 min at 95°C.

To test soluble complex formation, soluble protein extract (supernatant) was added to MagStrep ‘‘type3’’ XT beads (IBA Lifesciences) or anti-HA magnetic beads (MedChemExpress) and incubated in an anaerobic chamber for 2 h at room temperature with shaking. The beads were then washed three times using 500 µl of 100 mM Tris-HCl, 150 mM NaCl (pH 8.0) for MagStrep ‘‘type3’’ XT beads, or 137 mM NaCl, 2.7 mM KCl, 8 mM Na_2HPO_4, 2 mM KH_2PO_4, 0.05% Tween 20 (pH 7.4) for anti-HA magnetic beads. Soluble NifEN protein complex was eluted by addition of 50 µl of 2x Laemmli buffer and subsequent heating for 10 min at 95°C.

NifEN expression, solubility and complex formation was analyzed by SDS-PAGE and immunoblotting.

NifEN sequence and structure analysis. For primary sequence alignments, multiple sequence alignments were performed using the Clustal Omega Multiple Sequence Alignment online tool (https://www.ebi.ac.uk/Tools/msa/clustalo/) (11). The natural NifEN fusion proteins from *A. variabilis* and *G. metallireducens* were first aligned to the *A. vinelandii* NifE and NifN protein sequences to determine which parts

within the natural fusions were relevant for alignment with the *A. vinelandii* NifE and NifN sequences. For structural alignments, the individual NifE and NifN structures were obtained using Alphafold (12, 13). The structures were fitted to the *A. vinelandii* NifEN structure (14) using the MatchMaker function of the Structure Comparison tool in UCSF Chimera (v. 1.16, https:// www.rbvi.ucsf.edu/chimera) (15). Matching was iterated by pruning long atom pairs until no pairs exceeded 2.0 Å, and the root-mean-square deviation (RMSD) obtained for the pruned atom pairs was used determine the structural similarity between the modelled NifEN variant and the $NifEN^{Av}$. Unpaired t-tests were used to test for structural similarity between complementing and non-complementing NifE or NifN variants, respectively, with the corresponding *A. vinelandii* subunit.

Protein and Fe measurements of isolated NifEN proteins. The NifEN protein concentrations were measured using the BCA protein assay (PIERCE) modified with iodoacetamide supplementation to eliminate the interfering effect from presence of DTH in the protein sample (16). Unless specified differently, the Fe content of the purified NifEN proteins was determined by a colorimetric method (17). The Fe content of NifEN proteins purified from *A. vinelandii* was determined by inductively coupled plasma mass spectrometry (ICP-MS) at the Metal Analysis Service Center of Virginia Tech (Table S5).

SDS-PAGE, Coomassie staining and immunoblot analysis. Proteins were separated by SDS-PAGE and then stained using Coomassie brilliant blue R-250 (Sigma) or prepared for immunoblotting by transfer to Protran Premium 0.45 µm nitrocellulose membranes (GE Healthcare). Membranes for immunoblotting were

first stained with Ponceau S (Sigma) to confirm efficient transfer and similar loading, then blocked by shaking in 5% non-fat milk in TBS-T (20 mM Tris-HCl (pH 7.5), 150 mM NaCl, 0.02% Tween-20) for 1 h at room temperature. Membranes were incubated with primary antibodies overnight at 4°C. The antibodies used and their dilution were as follows: polyclonal $NifU^{Av}$ (used at 1:2,000 in 5% BSA) and $NifS^{Av}$ (used at 1:1,000 in 5% BSA) antibodies, Strep-tag antibody (GenScript #A01737, used at 1:5,000 in 5% BSA), HA-tag antibody conjugated to peroxidase (Roche #12013819001, used at 1:500 in 5% BSA) or unconjugated HA-tag antibody (Roche #11867423001, used at 1:2,000 in 5% BSA). The membranes were incubated with secondary antibodies (Sigma) prepared at 1:20,000 in TBS-T supplemented with 2% non-fat milk for 2 h at room temperature. Enhanced chemiluminescence was used to detect proteins and the membranes were digitally developed using an iBright FL1000 system (ThermoFisher).

Peptide mass fingerprinting. Protein purity was assessed by SDS-PAGE analysis, and peptide mass fingerprinting was used for protein identification. For this, Coomassie blue-stained protein bands were sliced with a sterile scalpel blade and transferred to an Eppendorf tube containing Milli-Q water. Mass spectrometry analysis was performed by the Proteomic Unit at Universidad Complutense de Madrid.

Statistical analysis. Statistical analyses such as linear regression and unpaired t-tests were performed using Prism 6 (GraphPad). Technical replicates signify individual vials containing protein from a single purification procedure, or individual vials containing cells originating from the same culture. Biological

replicates signify individual vials containing protein from separate purification procedures, or individual vials containing cells originating from different cultures.

SUPPLEMENTARY FIGURES

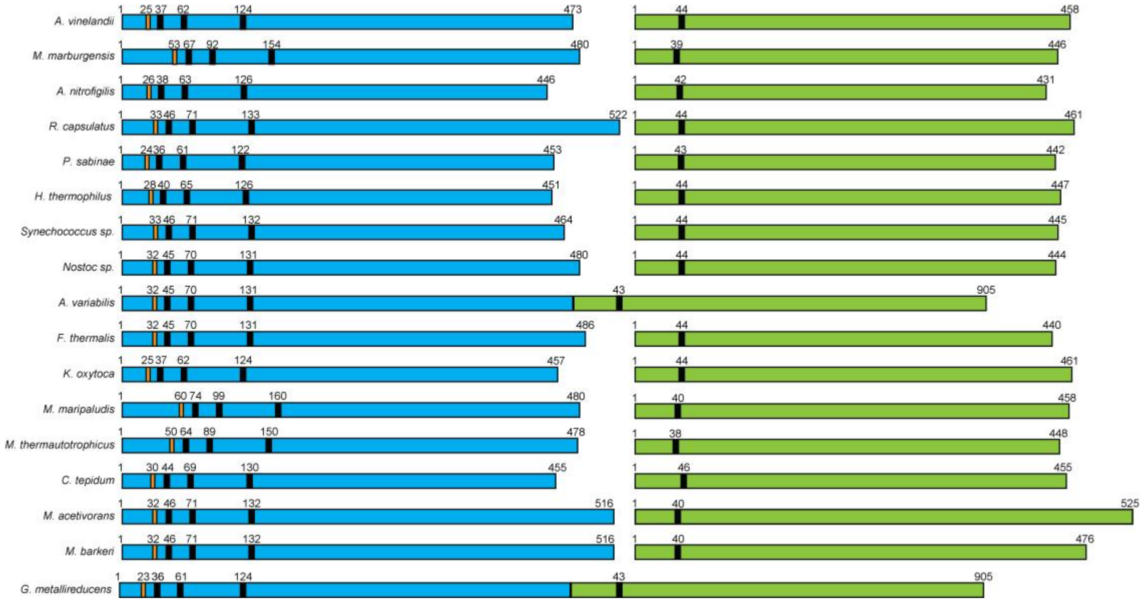

Figure S1. Alignment of NifE and NifN proteins depicting conserved cluster-binding Cys residues. NifE and NifN are represented as blue and green rectangular boxes, respectively. The ligands for binding the permanent [4Fe-4S] clusters (corresponding to Cys^{37}, Cys^{62} and Cys^{124} in the *A. vinelandii* NifE, and Cys^{44} in the *A. vinelandii* NifN) are marked in black. The putative ligand to the FeMo-co precursor (Cys^{25} in the *A. vinelandii* NifE) is marked in orange.

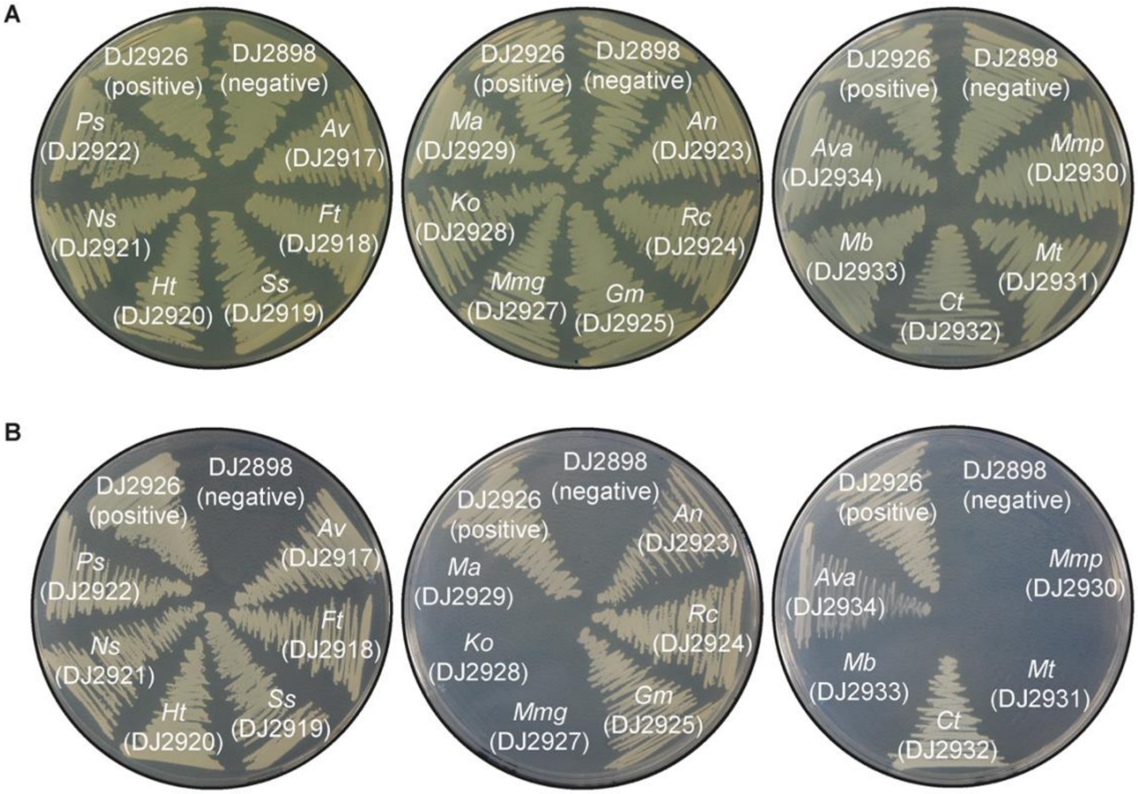

Figure S2. *In vivo* functionality of the 17 NifEN candidates in *A. vinelandii*. (A) *A. vinelandii* strains harboring the different *nifEN* genes were cultured for five days in solid Burk’s media supplemented with 13 mM ammonium acetate as the fixed the nitrogen source or (B) under diazotrophic conditions in nitrogen-free solid Burk’s media supplemented with arabinose. Note that the left plate in (B) is also presented in Fig. 2A. *Av, A. vinelandii*; *Ft*, *F. thermalis*; *Ss*, *Synechococcus sp. JA-2-3B'a*; *Ht*, *H. thermophilus; Ns*, *Nostoc sp.*; *Ps*, *P. sabinae*; *An*, *A. nitrofigilis*; *Rc*, *R. capsulatus*; *Gm*, *G. metallireducens*; *Mmg*, *M. marburgensis*; *Ko*, *K. oxytoca; Ma*, *M. acetivorans*; *Mmp*, *M. maripaludis*; *Mt*, *M. thermoautotrophicus*; *Ct*, *C. tepidum*; *Mb*, *M. barkeri*; *Ava*, *A. variabilis*. Refer to Table S2 for further details. Note that *An* (DJ2923) and *Gm* (DJ2925) had low values of ethylene production (Fig. 2B) while no apparent

differences in growth are observed in solid media after five days. These differences are a result of the culture method used, namely days of growth in solid media versus hours of growth in liquid media.

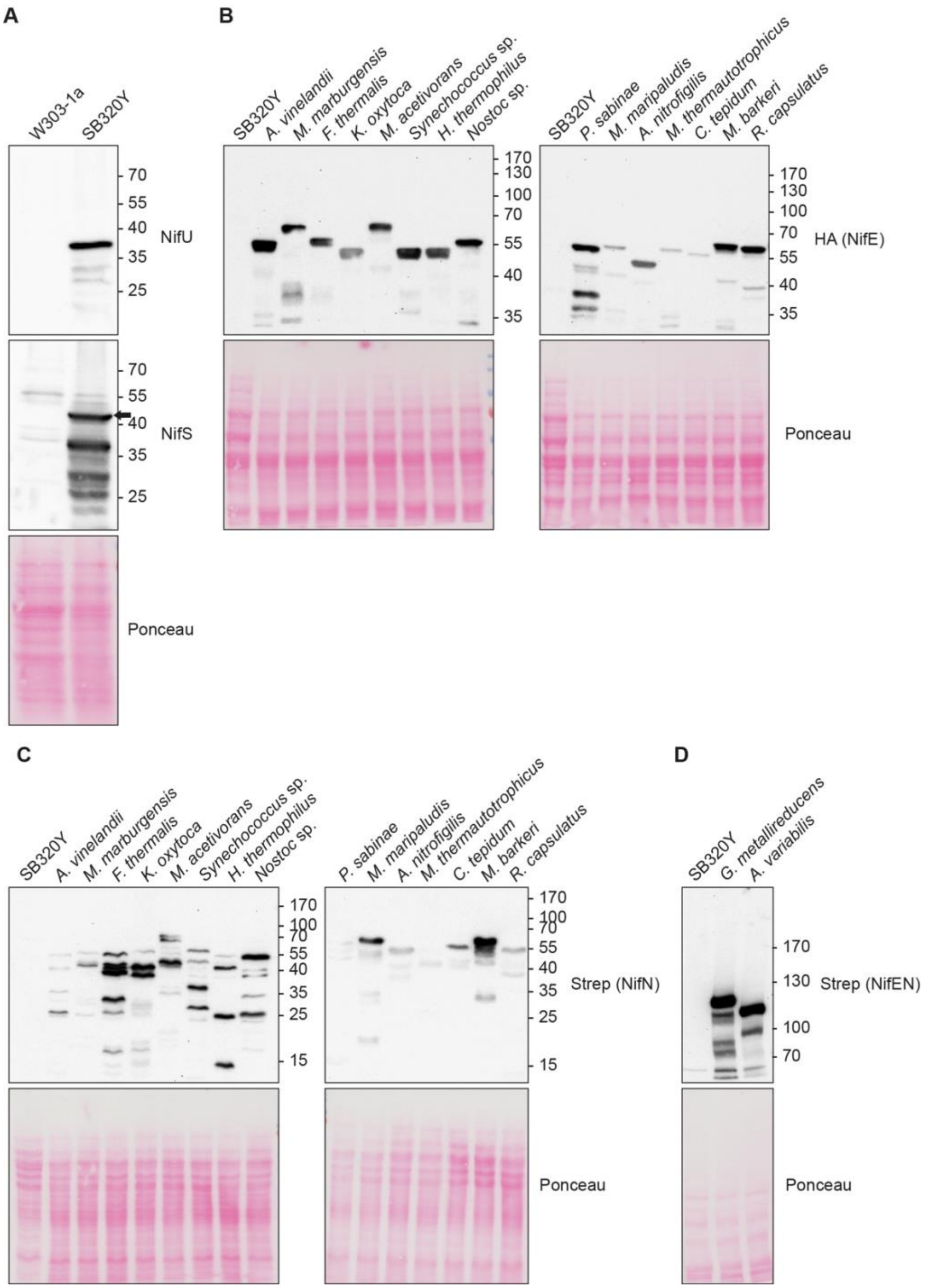

Figure S3. Expression of NifEN variants in *S. cerevisiae*. (A) Immunoblot analysis of total protein extracts showing the expression of $NifU^{Av}$ and $NifS^{Av}$ in yeast strain

SB320Y used as parental strain for the NifEN library. The black arrow points to full-length NifS ^{Av} (51.3 kDa), where the faster migrating polypeptides likely represent NifS degradation products. Accumulation of HA-NifE (B), NifN-SS-tag (C) and TS-tag NifEN (D) in total protein yeast extracts. Molecular weight markers (kDa) and antibodies used are indicated.

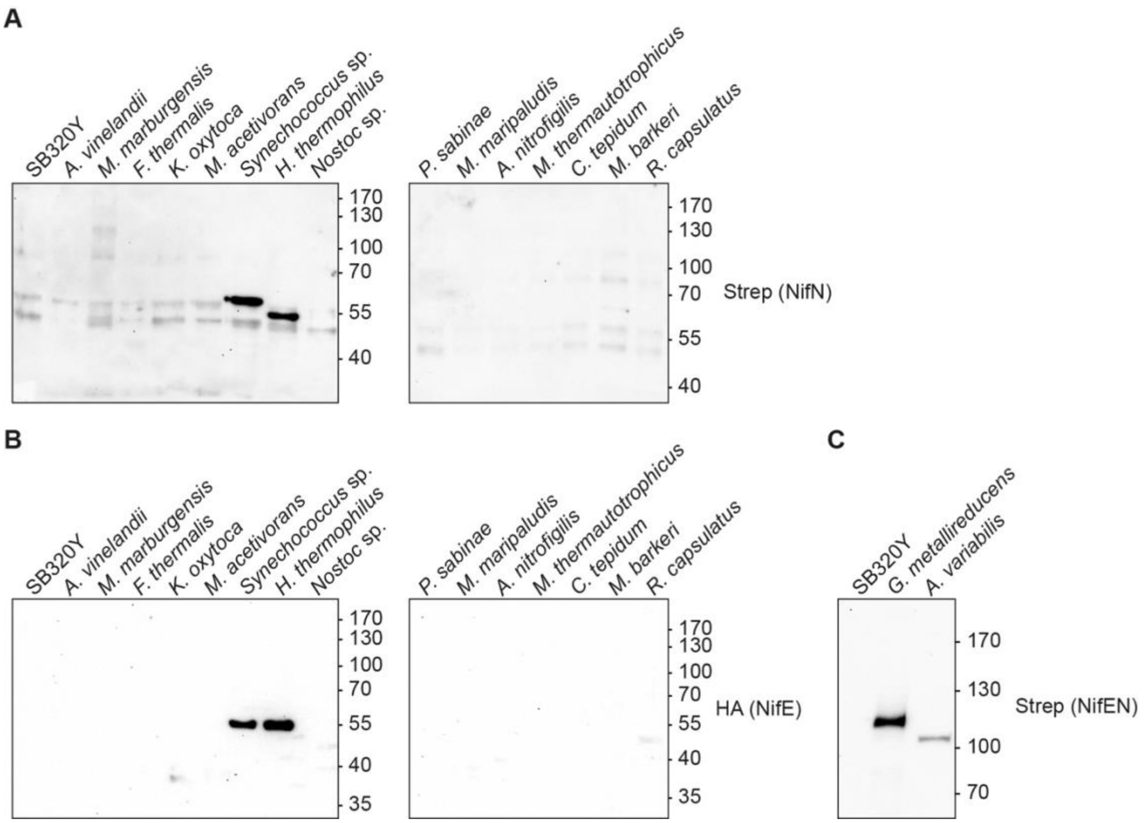

Figure S4. Co-purification and purification experiments to identify soluble NifEN variants accumulating in the mitochondria of aerobically cultured *S. cerevisiae*. Immunoblot analysis to detect SS-NifN subunits in HA-NifE co-purifications (A), HA-NifE subunits in NifN-SS co-purifications (B), and NifEN fusion proteins in TS-NifEN purifications. The presented immunoblots are representative of three independent experiments. Molecular weight markers (kDa) and antibodies used are indicated.

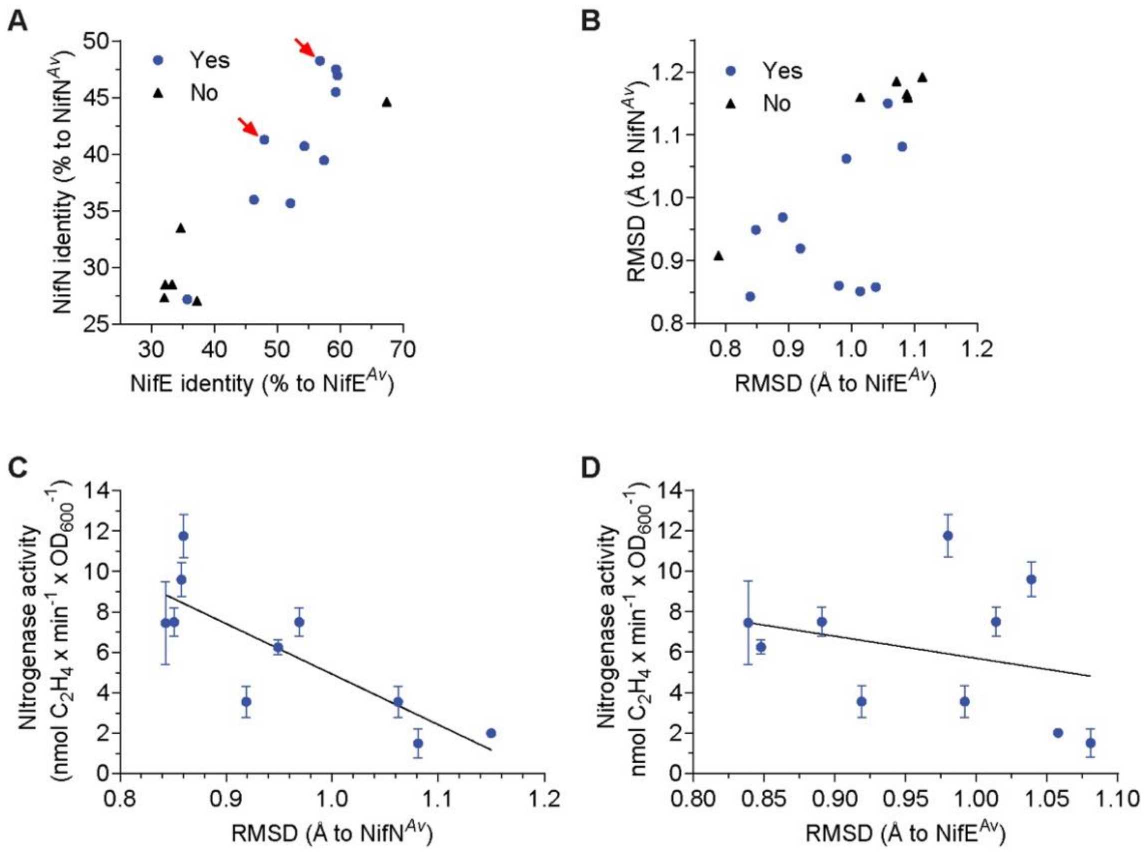

Figure S5. Sequence and structural divergence analysis of NifEN proteins in the library.

(A) Identity of NifE and NifN primary sequences to the respective *A. vinelandii* sequence and their capacity to restore diazotrophic growth in the *A. vinelandii* mutant strain DJ2898. The NifE and NifN sequences were analyzed using Clustal Omega. Blue dots represent NifEN proteins that restored diazotrophic growth, black triangles symbolize those variants that did not restore diazotrophic growth. The two natural NifEN fusion proteins are highlighted with red arrows. (B) Structural alignments of the NifE and NifN subunits of the NifEN library against the *A. vinelandii* NifE and NifN. The root-mean-square deviation (RMSD) between the predicted structures

obtained using Alphafold, and NifE and NifN in the *A. vinelandii* NifEN are plotted. Blue dots indicate NifEN variants that restored diazotrophic growth in DJ2898 while black triangles indicate those that did not. (C, D). The RMSD between the predicted NifN (C) or NifE (D) structures and the corresponding *A. vinelandii* NifN or NifE in *A. vinelandii* NifEN (seen in panel B) are plotted against the measured nitrogenase activity in the strains expressing the corresponding NifEN (Fig. 2B). The lines show linear regression analyses (panel C, $R^2 = 0.64$, $p < 0.0001$; and panel D, $R^2 = 0.08$, $p = 0.2350$).

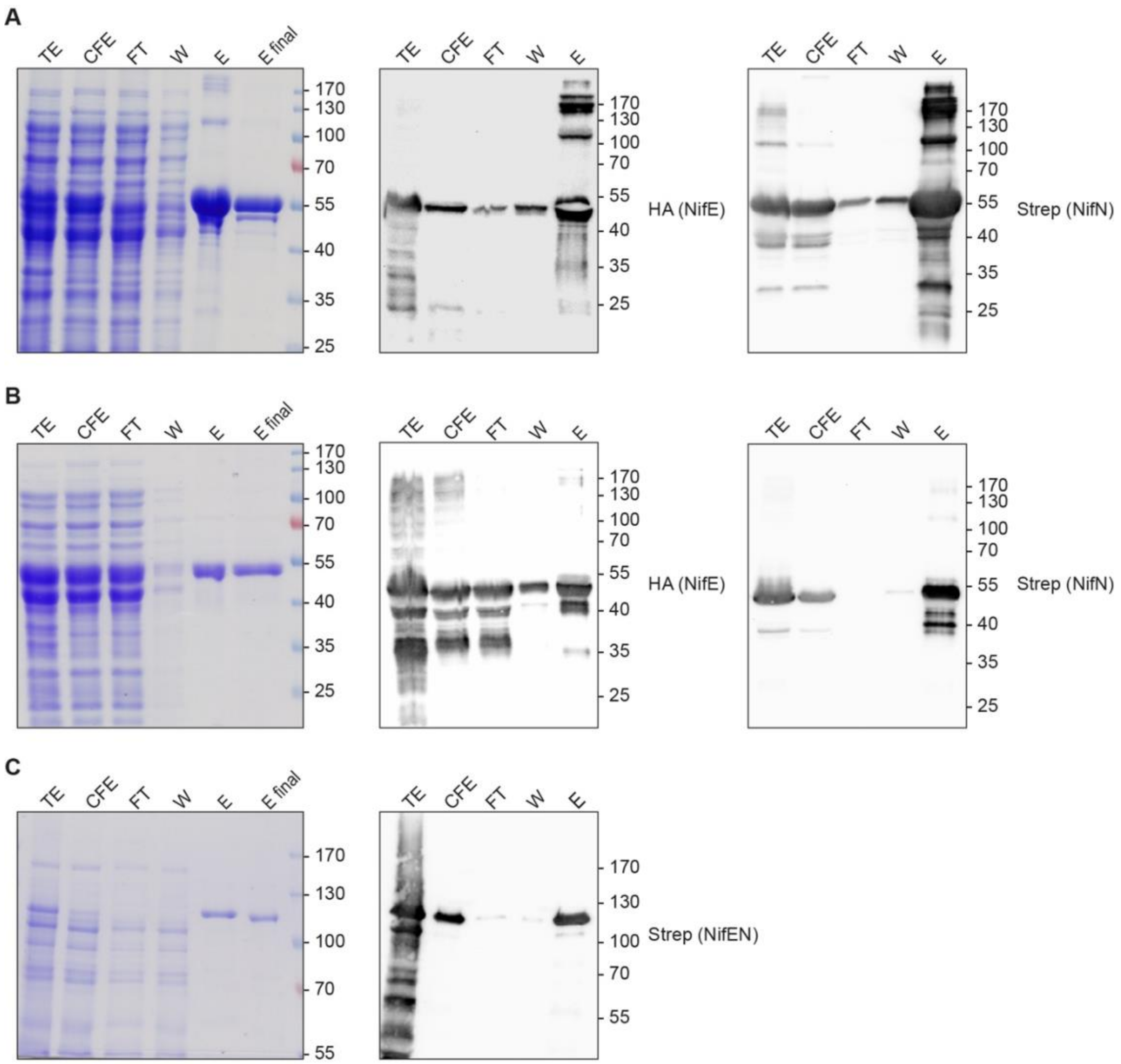

Figure S6. STAC purification of NifEN variants expressed in *E. coli* cells. *Ec*NifEN^{Ss} (A), *Ec*NifEN^{Ht} (B) and *Ec*NifEN^{Gm} (C) were isolated from *E. coli* cells co-expressing NifU^{Av} and NifS^{Av}. Fractions were analyzed by SDS-PAGE followed by Coomassie staining and immunoblot analysis, TE = total extract, CFE = soluble cell-free extract, FT = flow through fraction, W = wash fraction, E = elution fraction, E final = final protein fraction after desalting and concentration. Molecular weight markers (kDa) and antibodies used are indicated.

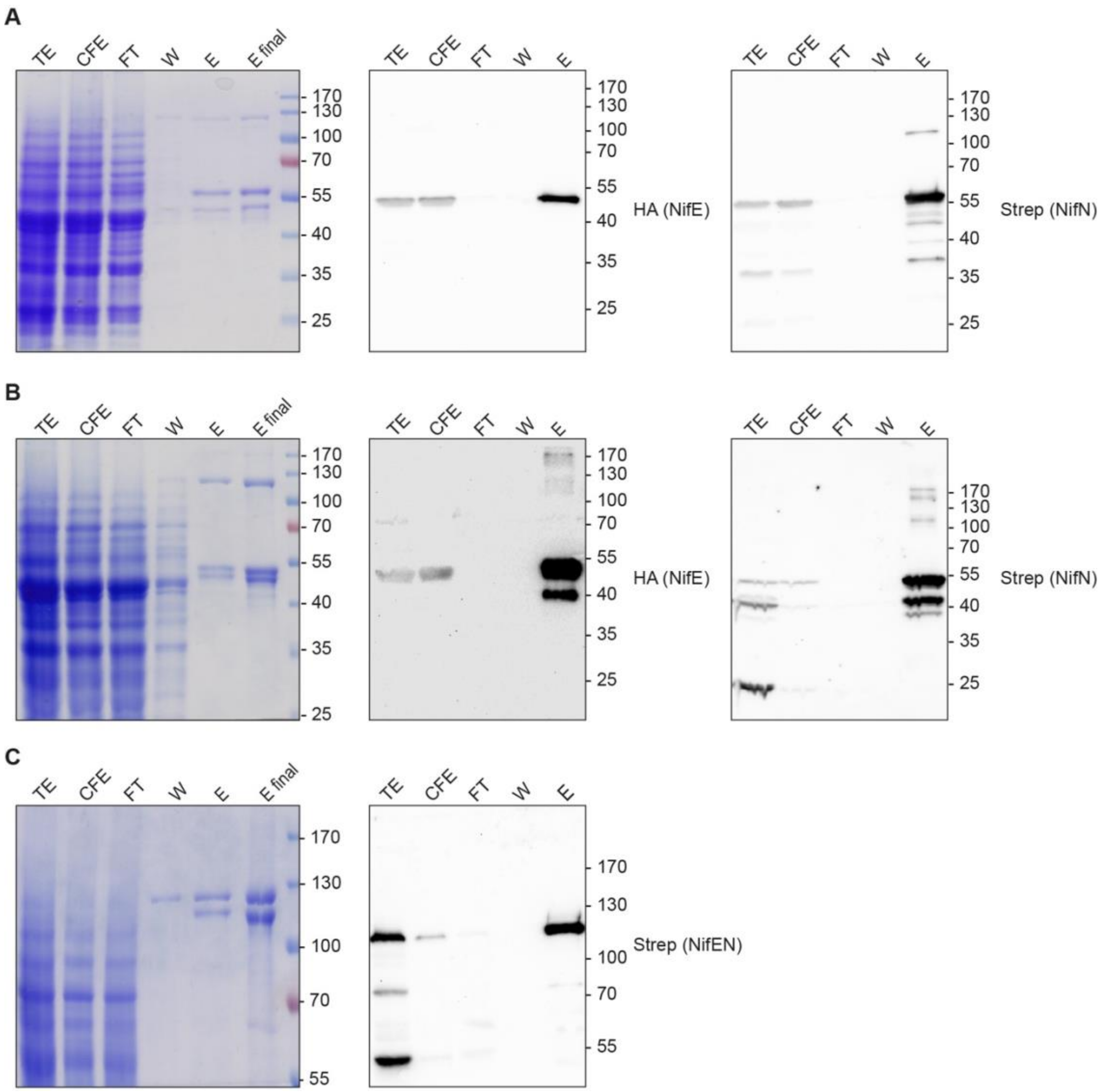

Figure S7. STAC purification of NifEN variants expressed in aerobic cultures of *S. cerevisiae.* *Sc*NifEN^{Ss} (A), *Sc*NifEN^{Ht} (B) and *Sc*NifEN^{Gm} (C) were isolated from yeast cells co-expressing NifU^{Av} and NifS^{Av}. Fractions were analyzed by SDS-PAGE followed by Coomassie staining and immunoblot analysis, TE = total extract, CFE = soluble cell free extract, FT = flow through fraction, W = wash fraction, E = elution

fraction, E final = final protein fraction after desalting and concentration. Molecular weight markers (kDa) and antibodies used are indicated.

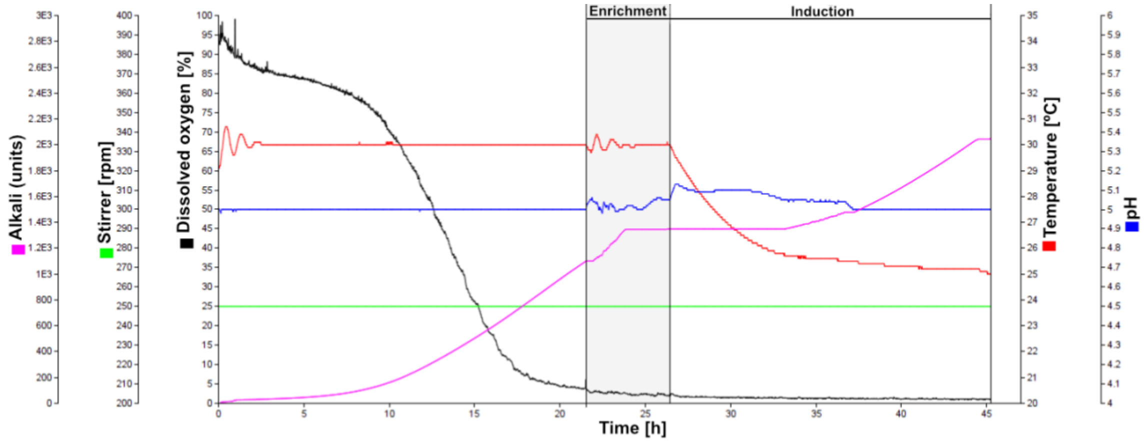

Figure S8. Typical batch fermentation process of the *S. cerevisiae* strain expressing *Sc*NifEN^{Gm} (KD37Y)**.** The following parameters were monitored during the process: dissolved oxygen (black), temperature (red), pH (blue) and alkali addition (pink) (to maintain constant pH). Stirring (green) was maintained at 250 rpm throughout the process. Culture turbidity (OD_{600}) was measured manually throughout the process. In the initial phase, the culture was grown in a synthetic drop-out (SD) medium supplemented with amino acids containing 3.3% glucose. When the dissolved oxygen dropped to below ~5%, the culture was enriched (indicated in grey) (around 22 h). Dissolved oxygen remained close to 0% during the rest of the process. In the enrichment phase, rich medium (0.25% yeast extract, 0.25% bactopeptone, 0.25% bactotryptone and 2.5% sucrose) was added to the culture, supplemented with 25 mg/l ammonium iron (III) citrate, 0.225 mM iron (II) sulfate, 1.25 mM magnesium sulfate, 1.5 mM calcium chloride and vitamin solution. In the induction phase (the white area starting at 27 h), 2.25% galactose, 0.25% bactopeptone and 0.25% yeast

extract were added to induce Nif protein expression. The turbidity at the end of the process (45 h) was 22.5 (OD_{600}) and the cells were harvested.

REFERENCES

1. UniProt-Consortium. 2023. UniProt: the universal protein knowledgebase in 2023. Nucleic Acids Res 51:D523-D531. https://doi.org/10.1093/nar/gkac1052.

2. Koskela EV, Frey AD. 2015. Homologous recombinatorial cloning without the creation of single-stranded ends: exonuclease and ligation-independent cloning (ELIC). Mol Biotechnol 57:233-40. https://doi.org/10.1007/s12033-014-9817-2.

3. Dos Santos PC, Johnson DC, Ragle BE, Unciuleac MC, Dean DR. 2007. Controlled expression of *nif* and *isc* iron-sulfur protein maturation components reveals target specificity and limited functional replacement between the two systems. J Bacteriol 189:2854-62. https://doi.org/10.1128/jb.01734-06.

4. Goodwin PJ, Agar JN, Roll JT, Roberts GP, Johnson MK, Dean DR. 1998. The *Azotobacter vinelandii* NifEN complex contains two identical [4Fe-4S] clusters. Biochemistry 37:10420-8. https://doi.org/10.1021/bi980435n.

5. Dos Santos PC. 2011. Molecular biology and genetic engineering in nitrogen fixation, p 81-92. *In* Ribbe MW (ed), Nitrogen Fixation: Methods and Protocols. Humana Press, Totowa, NJ.

6. Strandberg GW, Wilson PW. 1968. Formation of the nitrogen-fixing enzyme system in *Azotobacter vinelandii*. Can J Microbiol 14:25-31. https://doi.org/10.1139/m68-005.

7. Fajardo AS, Legrand P, Paya-Tormo LA, Martin L, Pellicer Martínez MT, Echavarri-Erasun C, Vernede X, Rubio LM, Nicolet Y. 2020. Structural insights into the mechanism of the radical SAM carbide synthase NifB, a key nitrogenase cofactor maturating enzyme. J Am Chem Soc 142:11006-11012. https://doi.org/10.1021/jacs.0c02243.

8. Burén S, Pratt K, Jiang X, Guo Y, Jiménez-Vicente E, Echavarri-Erasun C, Dean DR, Saaem I, Gordon DB, Voigt CA, Rubio LM. 2019. Biosynthesis of the nitrogenase active-site cofactor precursor NifB-co in *Saccharomyces cerevisiae*. Proc Natl Acad Sci U S A 116:25078-25086. https://doi.org/10.1073/pnas.1904903116.

9. Gietz RD, Schiestl RH. 2007. Quick and easy yeast transformation using the LiAc/SS carrier DNA/PEG method. Nat Protoc 2:35-7. https://doi.org/10.1038/nprot.2007.14.

10. Burén S, Young EM, Sweeny EA, Lopez-Torrejón G, Veldhuizen M, Voigt CA, Rubio LM. 2017. Formation of nitrogenase NifDK tetramers in the mitochondria of *Saccharomyces cerevisiae*. ACS Synth Biol 6:1043-1055. https://doi.org/10.1021/acssynbio.6b00371.

11. Sievers F, Wilm A, Dineen D, Gibson TJ, Karplus K, Li W, Lopez R, McWilliam H, Remmert M, Soding J, Thompson JD, Higgins DG. 2011. Fast, scalable generation of high-quality protein multiple sequence alignments using Clustal Omega. Mol Syst Biol 7:539. https://doi.org/10.1038/msb.2011.75.

12. Jumper J, Evans R, Pritzel A, Green T, Figurnov M, Ronneberger O, Tunyasuvunakool K, Bates R, Zidek A, Potapenko A, Bridgland A, Meyer C, Kohl SAA, Ballard AJ, Cowie A, Romera-Paredes B, Nikolov S, Jain R, Adler J, Back T, Petersen S, Reiman D, Clancy E, Zielinski M, Steinegger M, Pacholska M, Berghammer T, Bodenstein S, Silver D, Vinyals O, Senior AW, Kavukcuoglu K, Kohli P, Hassabis D. 2021. Highly accurate protein structure prediction with AlphaFold. Nature 596:583-589. https://doi.org/10.1038/s41586-021-03819-2.

13. Varadi M, Anyango S, Deshpande M, Nair S, Natassia C, Yordanova G, Yuan D, Stroe O, Wood G, Laydon A, Zidek A, Green T, Tunyasuvunakool K, Petersen S, Jumper J, Clancy E, Green R, Vora A, Lutfi M, Figurnov M, Cowie A, Hobbs N, Kohli P, Kleywegt G, Birney E, Hassabis D, Velankar S. 2022. AlphaFold protein structure database: massively expanding the structural coverage of protein-sequence space with high-accuracy models. Nucleic Acids Res 50:D439-D444. https://doi.org/10.1093/nar/gkab1061.

14. Kaiser JT, Hu Y, Wiig JA, Rees DC, Ribbe MW. 2011. Structure of precursor-bound NifEN: a nitrogenase FeMo cofactor maturase/insertase. Science 331:91-4. https://doi.org/10.1126/science.1196954.

15. Pettersen EF, Goddard TD, Huang CC, Couch GS, Greenblatt DM, Meng EC, Ferrin TE. 2004. UCSF Chimera: a visualization system for exploratory research and analysis. J Comput Chem 25:1605-12. https://doi.org/10.1002/jcc.20084.

16. Hill HD, Straka JG. 1988. Protein determination using bicinchoninic acid in the presence of sulfhydryl reagents. Anal Biochem 170:203-208. https://doi.org/10.1016/0003-2697(88)90109-1.

17. Fish WW. 1988. Rapid colorimetric micromethod for the quantitation of complexed iron in biological samples. Methods Enzymol 158:357-64. https://doi.org/10.1016/0076-6879(88)58067-9.
